# Mini-Review: GSDME-Mediated Pyroptosis in Diabetic Nephropathy

**DOI:** 10.3389/fphar.2021.780790

**Published:** 2021-11-16

**Authors:** Wen Li, Jing Sun, Xiaoxi Zhou, Yue Lu, Wenpeng Cui, Lining Miao

**Affiliations:** Department of Nephropathy, The Second Hospital of Jilin University, Changchun, China

**Keywords:** pyroptosis, gasdermin, GSDME, caspase-3, diabetic nephropathy

## Abstract

Pyroptosis is a recently identified type of lytic programmed cell death, in which pores form in the plasma membrane, and cells swell, rupture, and then release their contents, including inflammatory cytokines. Molecular studies indicated that pyroptosis may occur *via* a gasdermin D (GSDMD) and caspase-1 (Casp1) -dependent classical pathway, a GSDMD and Casp11/4/5-dependent non-classical pathway, or a gasdermin E (GSDME) and Casp3-dependent pathway. Studies of animal models and humans indicated that pyroptosis can exacerbate several complications of diabetes, including diabetic nephropathy (DN), a serious microvascular complication of diabetes. Many studies investigated the mechanism mediating the renoprotective effect of GSDMD regulation in the kidneys of patients and animal models with diabetes. As a newly discovered regulatory mechanism, GSDME and Casp3-dependent pyroptotic pathway in the progression of DN has also attracted people’s attention. Z-DEVD-FMK, an inhibitor of Casp3, ameliorates albuminuria, improves renal function, and reduces tubulointerstitial fibrosis in diabetic mice, and these effects are associated with the inhibition of GSDME. Studies of HK-2 cells indicated that the molecular and histological features of secondary necrosis were present following glucose stimulation due to GSDME cleavage, such as cell swelling, and release of cellular contents. Therefore, therapies targeting Casp3/GSDME-dependent pyroptosis have potential for treatment of DN. A novel nephroprotective strategy that employs GSDME-derived peptides which are directed against Casp3-induced cell death may be a key breakthrough. This mini-review describes the discovery and history of research in this pyroptosis pathway and reviews the function of proteins in the gasdermin family, with a focus on the role of GSDME-mediated pyroptosis in DN. Many studies have investigated the impact of GSDME-mediated pyroptosis in kidney diseases, and these studies used multiple interventions, *in vitro* models, and *in vivo* models. We expect that further research on the function of GDSME in DN may provide valuable insights that may help to improve treatments for this disease.

## Introduction

There are several programmed cell death (PCD) pathways (Abbreviations in the manuscript are listed in Supplementary Table 1), and each pathway depends on different genes, signals, and cellular activities. Each of the PCD pathways removes functionally dispensable, infected, or potentially neoplastic cells. PCD is therefore an important component of homeostasis and also provides host defense against pathogens, cancer, and other pathologies (Bedoui et al., 2020). The major types of PCD are pyroptosis, apoptosis, and necroptosis (Wang and Kanneganti, 2021). Disturbances of extracellular or intracellular homeostasis that destroy innate immunity can lead to pyroptosis, also referred to as gasdermin (GSDM)-dependent programmed necrosis, the most recently defined type of PCD (Jorgensen and Miao, 2015). Pyroptosis is a form of PCD characterized by greatly increased inflammation, and it most commonly occurs after a serious infection, but can also occur in chronic diseases (Shi et al., 2017; Galluzzi et al., 2018).

Histological studies indicated that pyroptosis has some features of apoptosis and some features of necrosis. More specifically, cells undergoing pyroptosis develop pores in the cytoplasmic membrane, become swollen, and then rupture, leading to the release pro-inflammatory mediators, such as interleukin-1β (IL-1β) and interleukin-18 (IL-18) (Galluzzi et al., 2018). At the molecular level, pyroptosis is characterized by increased activity of the canonical pathway of caspase-1 (Casp1) and/or the non-canonical pathway of Casp4/5 (humans) and Casp11 (mice), as well as their downstream execution protein gasdermin D (GSDMD) (Kayagaki et al., 2015; Shi et al., 2015). A recent study by Rogers et al. found that Casp3 successfully induced apoptosis and then cleaved GSDME, leading to generation of an N-terminal fragment (N-GSDME) and induction of pyroptosis (Rogers et al., 2017). Wang et al. confirmed that Casp3 cleavage led to formation of N-GSDME, and this interacted with 4,5-diphosphate phosphatidylinositol (PI[4,5]P2), and that this was followed by the perforation of liposomes and release of their contents (Wang et al., 2017).

Therefore, Casp3, which has a well-established role in apoptosis (Cohen, 1997), also functions in pyroptosis by cleavage of GSDME. The cleavage of GSDME after treatment of cancer with chemotherapy agents provides a cross-talk between the pro-inflammatory process of pyroptosis and the anti-inflammatory process of apoptosis (Zheng and Li, 2020). Thus, GSDME may be regarded as a “molecular switch”, in that its cleavage status determines whether cells undergo pyroptosis or apoptosis. There is also evidence that the effects of GSDME are downstream and upstream of Casp3, in that it connects the endogenous and exogenous apoptosis pathways and it also increases activation of Casp3, thereby functioning in a positive feedback loop (Jiang et al., 2020). The expression of GSDME therefore controls whether cell death occurs *via* apoptosis or pyroptosis. In the presence of GSDME, some chemotherapy drugs induce Casp3 activation and lead to cell death *via* pyroptosis (Rogers et al., 2017; Wang et al., 2017) because pyroptosis is more rapid than apoptosis (Shi et al., 2015).

Pyroptosis functions in several different clinical pathologies, including type-2 diabetes. The ninth Edition of the International Diabetes Federation Diabetes Atlas reported in a worldwide survey that there will be 1.25 billion people with diabetes by 2030 and 1.50 billion by 2045 (Saeedi et al., 2019). Diabetic kidney disease (DKD) is a very serious microvascular complication of diabetes, and the incidence rate of DKD is also increasing worldwide (Harjutsalo and Groop, 2014; Huang et al., 2019). There is therefore a critical need for novel treatments of diabetic nephropathy (DN), because it is a primary cause of end-stage renal disease throughout the world (Podgórski et al., 2019). Increased renal inflammation and renal fibrosis play critical roles in the onset and progression of DN (Sabapathy et al., 2019; Liu et al., 2020).

Recent research confirmed that Z-DEVD-FMK, a tetrapeptide that inhibits Casp3, reduced albuminuria, renal dysfunction, and tubulointerstitial fibrosis in diabetic mice (Wen et al., 2020). The nephroprotective effects of Z-DEVD-FMK were presumably due to its inhibition of GSDME activation. Other *in vitro* studies reported the presence of molecular and morphological features of secondary necrosis in HK-2 cells that were stimulated by glucose, and this was accompanied by GSDME cleavage, swelling of cells, and release of cellular contents (Wen et al., 2020). It is therefore believed that disruption of Casp3/GSDME-dependent pyroptosis has potential as a treatment for DN. We examined therapies that focus on pyroptosis and the Casp3/GSDME signaling pathway in DN in this mini-review. We first compare pyroptosis with other forms of PCD, introduce the history of scientific studies of pyroptosis, and then describe the functions of proteins in the GSDM family, especially GSDME and describe the role of GSDME-mediated pyroptosis during DN.

## Comparison of Pyroptosis, Necroptosis, Ferroptosis, and Apoptosis

The development and homeostasis of tissues and organisms requires regulation of cell proliferation and the removal of cells that are unnecessary or harmful. This includes the removal of damaged cells that may undergo neoplastic transformation and of cells whose functions are controlled by pathogenic microbes to promote pathogen replication (Bedoui et al., 2020). Cell death can be broadly classified as PCD or non-PCD (Durand et al., 2011). The different types of PCD depend on the regulation of specific genes that encode signals or activities that remove functionally dispensable, infected, or potentially neoplastic cells (Bedoui et al., 2020). The major types of PCD are pyroptosis, necroptosis, ferroptosis, and apoptosis (Green, 2019; Bedoui et al., 2020); the first three types are considered lytic cell death, and apoptosis is considered non-lytic. These different forms of PCD can be distinguished by different initiators, effectors, and executioners (Wang and Kanneganti, 2021).

Pyroptosis is a type of lytic and inflammatory cell death whose main features are cytoplasmic membrane pore formation, cellular edema, membrane rupture, and the release of inflammatory factors (Galluzzi et al., 2018). Molecular studies reported that pyroptosis is driven by Casp1, Casp4, Casp5, and Casp11, as well as Casp3 (which functions as an executioner caspase in apoptosis). The canonical pathway of pryoptosis is characterized by activation of inflammasomes by pathogen-associated molecular patterns (PAMPs) or danger-associated molecular patterns (DAMPs). This leads to recruitment of Casp1, which promotes the dimerization and activation of these compounds. Activated Casp1 then leads to synthesis and secretion of multiple inflammatory factors, including IL-1β and IL-18 (Wang et al., 2019b). In contrast, the non-canonical pathway of pyroptosis depends on the function of Casp4/5 in mice (corresponding to Casp11 in humans), which recognize lipopolysaccharide (LPS) *via* the caspase activation and recruitment domains (CARDs) (Kayagaki et al., 2011; Shi et al., 2014; Kovacs and Miao, 2017). The canonical and non-canonical pathways of pyroptosis are both characterized by inflammatory caspases that activate the pore-formation protein GSDMD (Xu et al., 2018). Recent studies confirmed that Casp3 also functions in pyroptosis (Jiang et al., 2020; Shen et al., 2021). In particular, Casp3 can be activated by the intrinsic apoptosis initiator (Casp9) or by the extrinsic apoptosis initiator (Casp8). Upon activation, Casp3 induces pyroptosis *via* cleavage of GSDME.

Necroptosis is another form of lytic PCD that is defined by the destruction of the cytoplasmic membrane and cellular swelling (Tonnus et al., 2019). Numerous signaling molecules can induce necroptosis *via* death receptors or pathogen recognition receptors (PRRs) (Galluzzi et al., 2014; Choi et al., 2019). Three proteins have important functions in necroptosis: receptor-interacting protein kinase 1 (RIPK1), RIPK3, and mixed lineage kinase domain-like (MLKL) (Belavgeni et al., 2020). Notably, necroptosis is considered independent of capsases (Conrad et al., 2016). Instead, upon initiation by receptors, necroptosis is mediated by RIPK1 or RIPK3-induced MLKL phosphorylation, followed by the formation of MLKL pores (Wang and Kanneganti, 2021). Interestingly, necroptosis usually occurs when the extrinsic pathway of apoptosis is non-functional (Frank and Vince, 2019). Recent research found that the occurrence of necroptosis was inhibited by Casp8 (Nailwal and Chan, 2019), which can inactivate RIPK1 *via* proteolytic cleavage (Newton et al., 2019). However, viral infections can inhibit Casp8, thus driving necroptosis (Belavgeni et al., 2020).

Ferroptosis, a newly described form of PCD, is driven by lipid peroxidation and depends on the availability of iron and the generation of reactive oxygen species (ROS) (Galluzzi et al., 2018). A key feature of ferroptosis is intracellular overload iron and catalysis of lipid peroxidation (Belavgeni et al., 2020). Ferroptosis differs from the other forms of PCD in that it is characterized by small dysmorphic mitochondria with fewer cristae, a ruptured outer membrane, and a condensed inner membrane (Wang et al., 2020a; Sun et al., 2020), Ferroptosis functions in a variety of biological processes, including oxidative stress, iron metabolism, and lipid metabolism (Qiu et al., 2020). In return, the levels of iron, ROS, glutathione (GSH), and related regulatory molecules can regulate ferroptosis (Qiu et al., 2020). Ferroptosis occurs mainly due to the disruption of endogenous cellular antioxidant defense systems. Glutathione peroxidase 4 (GPX4), is the only glutathione peroxidase that affects ferroptosis, in that it functions as an endogenous inhibitor of this form of PCD (Ursini et al., 1982). In particular, GPX4 catalyzes the reduction of GSH, thereby limiting lipid peroxidation and ferroptosis (Imai et al., 2017).

Apoptosis is the best known form of PCD and was first described in 1972, although many earlier studies described several of the key feature of this form of PCD (Kerr et al., 1972). Unlike the other types of PCD, apoptosis is an active series of steps that occurs during normal physiological processes, such as development and aging, to maintain homeostasis; Apoptosis also occurs during pathological states, such as when cells are injured by a disease or toxic agents (Norbury and Hickson, 2001). Histologically, apoptosis is characterized by shrinkage and pyknosis of cells, condensation of chromatin, fragmentation of nuclei, and the formation of apoptotic bodies and cytoskeletal disintegration, but no obvious histological changes in the mitochondria (Elmore, 2007; Li et al., 2020c).

Apoptosis may occur *via* an intrinsic pathway (initiation by intracellular signals) or an extrinsic pathway (initiation by extracellular signals), and each pathway requires distinct caspases (Elmore, 2007). The intrinsic (mitochondrial) pathway is driven by numerous intracellular events, such as DNA damage, ROS formation, endoplasmic reticulum stress, and others. These stimuli then produce a change in the *trans*-membrane potential of the mitochondria, leading to the release of pro-apoptotic proteins, especially cytochrome c (Cyto C), into the cytoplasm (Kiraz et al., 2016). Subsequently, Cyto C interacts with Apf-1 to activate Casp9, which then cleaves pro-Casp3 into Casp3, the active form which functions in the execution phase (Li et al., 1997).

The extrinsic pathway of apoptosis begins upon activation of the plasma membrane-bound death receptor and the dependence receptor. The death receptor is activated by binding to ligand(s), and the dependence receptor is activated when the levels of associated ligands decline below a certain threshold (Galluzzi et al., 2018). Once triggered, pro-Casp8 is recruited and processed to Casp8 (Muzio et al., 1996), which then activates the downstream executioners, Casp3 and Casp7 (Stennicke et al., 1998; Twiddy et al., 2006), leading to a series of histological and molecular changes.

It is important to note that pyroptosis, necroptosis, ferroptosis, and apoptosis have different initiators, effectors, and executioners (Wang and Kanneganti, 2021), and this is associated with differences in their histological and biochemical features (Belavgeni et al., 2020; Li et al., 2020c). All types of PCD except apoptosis are passive processes (Norbury and Hickson, 2001). In addition, the different modes of PCD may play different roles in immune defense. For example, pyroptosis is primarily a cellular reaction to potential damage or insult, such as pathogen invasion (Frank and Vince, 2019). Necroptosis is critical in the host response to infections, especially by viruses (Brault and Oberst, 2017), and it functions as a backup mechanism of cell death and defense when apoptosis is blocked, such as during pathogen infection (Frank and Vince, 2019). Ferroptosis is a form of cellular antioxidant defense against oxidative stress and it functions in a variety of diseases, not just infectious diseases (Kuang et al., 2020). Apoptosis is regarded as a mechanism of self-protection that eliminates dead or damaged cells that form during the physiological process of aging (Tomei and Umansky, 1998; Argüelles et al., 2019). Apoptosis also has an important function in the suppression of tumor growth, and some anticancer drugs function by stimulation of apoptosis (Kaczanowski, 2016). Although these distinct types of PCD have different functions in different physiological states and in a host’s response to external damage, there is clear evidence of crosstalk or interactions (Wang and Kanneganti, 2021), and some of the same proteins function in multiple types of PCD. For instance, Casp3 functions in apoptosis and GSDME-mediated pyroptosis (Jiang et al., 2020). Casp8 initiates the extrinsic apoptosis pathway, and can also inhibit necroptosis (Newton et al., 2019). In addition, the same stimulant may induce different types of PCD, depending on its intensity or action time (Bedoui et al., 2020).

## Historical Perspective of Research on Pyroptosis

The term pyroptosis derives from the Greek words ‘pyro’ (fever) and ‘ptosis’ (falling) (Cookson and Brennan, 2001). This newly identified form of PCD is characterized by the formation of large pores in the plasma membrane, followed by cell swelling and rupture (Galluzzi et al., 2018). Before this word was adopted, this form of PCD was described by Friedlander in 1986, who treated primary mouse macrophages with anthrax toxin and reported the rapid release of the cell contents (Friedlander, 1986). In 1992, researchers first described this inflammatory PCD as a type of apoptosis in their studies of infection by Shigella flexneri (Zychlinsky et al., 1992). Five years later, Hilbi et al. discovered that Shigella dysenteriae activated Casp1 in host cells (Hilbi et al., 1997). In 1999, Hersh et al. confirmed that knocking out Casp1 blocked Salmonella-induced PCD (Hersh et al., 1999).

The year 2001 was an important turning point in our understanding of pyroptosis. Boise et al. observed macrophage death induced by bacterial infection, and identified a type of PCD that different from apoptosis, and named this pathway “caspase-1-dependent programmed necrosis” (Boise and Collins, 2001; Cookson and Brennan, 2001). Cookson et al. then proposed using the word “pyroptosis” for this proinflammatory mode of PCD (Cookson and Brennan, 2001). In 2015, researchers identified the GSDMD protein and identified it as the executioner of pyroptosis; they also reported that pyroptosis is auto-inhibited in normal and healthy cells, but that cleavage of GSDMD by Casp1 and Casp4/5 (Casp11 in humans) activated pyroptosis (Kayagaki et al., 2015; Shi et al., 2015). Subsequent research identified other proteins in the GSDM family (GSDMA, GSDMB, GSDMC, GSDME, and DFNB59) and confirmed their roles in membrane perforation and induction of pyroptosis (Martirosyan and Gorvel, 2013; Ding et al., 2016). In 2017, Rogers et al. reported that activated Casp3 induced apoptosis and then cleaved GSDME into N-GSDME, and this induced pyroptosis (Rogers et al., 2017). Wang et al. confirmed that N-GSDME interacted with PI(4,5)P2, and this led to the perforation of liposomes and loss of their phospholipids in a Casp3-mediated process (Wang et al., 2017). Therefore, to emphasize the role of multiple proteins in the GSDM family, Feng Shao and others characterized pyroptosis as “GSDM family-mediated programmed necrosis” (Shi et al., 2017).

**FIGURE 1:**
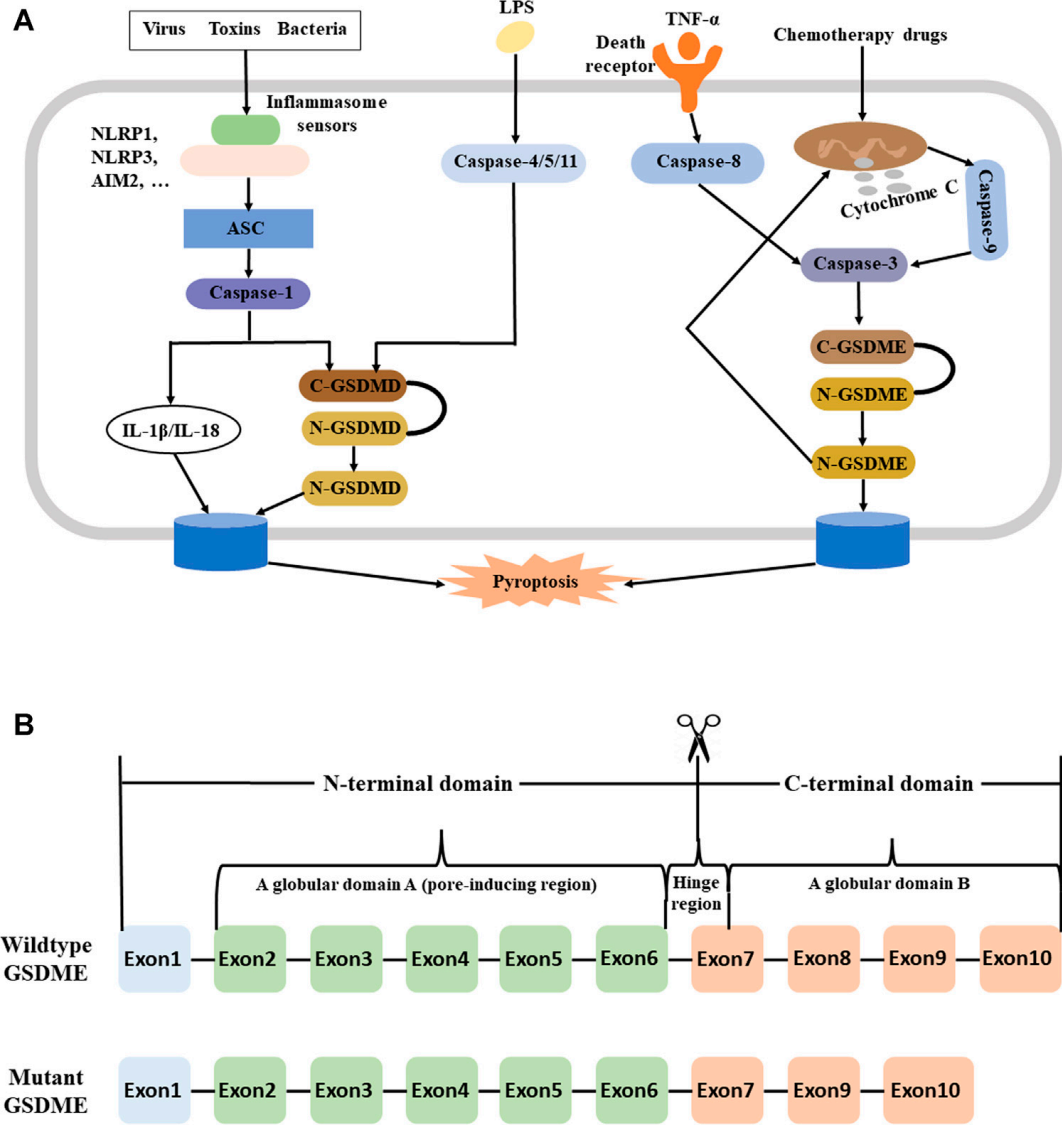
**(A)** Pyroptosis may occur *via* a GSDMD/Casp1-dependent canonical pathway, a caspase-11 (4/5) -dependent non-canonical pathway, and a Casp3/GSDME pathway. **Left:** In the canonical pathway, viruses, toxins (including DAMPs and PAMPs), or bacteria bind to inflammasome sensors, which then binds to an initiator protein (NLRP1, NLRP3, AIM2, … ). This leads to activation of an adaptor protein (ASC), an executioner caspase (typically a Casp1 initiator protein such as NLRP3 or AIM2), and then an executioner caspase (typically Casp1). Once activated, Casp1 induces the proteolysis of substrate proteins, including GSDMD and the pro-inflammatory cytokines IL-1β and IL-18, followed by membrane perforation and pyrolysis. **Middle:** In the noncanonical pathway, intracellular LPS or toxins activate Casp11 in mice (Casp4/5 in humans). This leads to the same cascade of reactions as in the canonical pathway. **Right:** In the Casp3/GSDME pathway, a death receptor or the mitochondrial pathway activates Casp3. This leads to cleavage of GSDME to N-GSDME, membrane perforation, and pyroptosis. The generation of N-GSDME also permeabilizes the mitochondrial membrane, leading to release of cytochrome C and induction of Casp3-mediated apoptosis, a positive feedback loop; **(B)** Structure of the wildtype and mutant GSDME gene. The wildtype *GSDME* has 10 exons and three domains: globular domain A (exon 2–6), a hinge region (exon 6–7), and globular domain B (exon 7–10). The hinge region differs in length and sequence among different *GSDME* variants. In wildtype, the N-terminal domain (exons 2–6) is responsible for production of plasma membrane pores and subsequent pyroptosis, and is only active upon cleavage from the C-terminal domain. However, individuals with *GSDME*-mediated hearing loss have a deletion of exon eight and protein truncation, leading to spontaneous formation of plasma membrane pores and pyroptosis. Abbreviations: GSDMD, gasdermin D; Casp3, caspase-3; GSDME, gasdermin E; DAMPs, danger-associated molecular patterns; PAMPs, pathogen-associated molecular patterns; NLRP1, NACHT, LRR, and PYD domains-containing protein 1; NLRP3, NACHT, LRR, and PYD domains-containing protein 3; AIM2, absent in melanoma 2; ASC, apoptosis-associated speck-like protein containing a caspase recruitment domain; IL-1β/IL-18, interleukin-1β/interleukin-18; LPS, lipopolysaccharide; N-GSDME, N-terminal fragment of gasdermin E.

In 2018, Kambara et al. discovered that neutrophil elastase (NE) promoted the cleaveage of GSDMD, and this led to a process they called “neutrophil pyroptosis” (Kambara et al., 2018). Also in 2018, the Nomenclature Committee on Cell Death (NCCD) defined pyroptosis as a form of regulated cell death (RCD) that was characterized by pore formation in the plasma membrane due to the actions of proteins in the GSDM family (Galluzzi et al., 2018). Many recent studies have described the function of pyroptosis during the onset and progression of multiple diseases or pathological states, such as cancer, inflammation, and fibrosis (Aglietti and Dueber, 2017; Kovacs and Miao, 2017; Shi et al., 2017; Xia et al., 2020). Figure 1A summarizes the functions of different signals, molecules, and pathways in pyroptosis.

Pyroptosis is now accepted as a pro-inflammatory form of PCD that functions as a host defense mechanism against invading pathogens and in response to other types of cellular injury (Aglietti and Dueber, 2017). Initial studies suggested that pyroptosis only occurred in immune cells (macrophages, monocytes, dendritic cells, and T cells) (Schroder and Tschopp, 2010; Jorgensen et al., 2017), and that stimulation of these cells by pathogens or their products led to pyroptosis due to the increased activity of pro-inflammatory caspases (Casp1 in humans and mice; Casp4/5 in humans, and Casp11 in mice) (Shi et al., 2014; Yuan et al., 2016). The original descriptions of pyroptosis thus characterized the Casp1-dependent classical pathway and the Casp4/5/11-dependent non-classical pathway.

Inflammasomes, regarded as components of the innate immune system, have a key role in the classical pathway of pyroptosis (Martinon et al., 2002). These large multiprotein complexes have a pattern recognition receptor (PRR), which function as an initiator protein (Ketelut-Carneiro and Fitzgerald, 2020). The protein composition of inflammasomes varies. The major PRRs are nucleotide-binding oligomerization domain and leucine-rich repeat-containing receptors (NLRs) (Song and Li, 2018). However, absent in melanoma 2 (AIM2), interferon-inducible protein 16 (IFN16), and pyrin (marenostrin, MEFV) also function as PRRs (Bedoui et al., 2020), Notably, inflammasomes also contain apoptosis-associated speck-like protein containing a caspase recruitment domain (ASC), which functions as an adaptor protein, and an executioner caspase (typically Casp1) (Martinon et al., 2002).

The activation of pyroptosis begins when the initiator directly or indirectly detects microbial ligands, followed by Casp1 activation (Orning et al., 2019). This induces proteolysis of substrate proteins, including GSDMD, and activates several pro-inflammatory cytokines, includingIL-1β and IL-18. However, Casp11 can also mediate non-canonical activation of inflammasomes. This non-canonical pathway acts on GSDMD without the NLR sensor and adaptor proteins that function in the canonical pathway. LPS or host-derived oxidized phospholipids activate Casp11 in mice (corresponding to Casp4/5 in humans) (Kayagaki et al., 2011; Hagar et al., 2013; Kayagaki et al., 2013; Shi et al., 2014; Zanoni et al., 2016). GSDM functions as the effector protein in the canonical and non-canonical pathways, and its cleavage by pro-inflammatory caspases at Asp275 in humans (Asp276 in mice) leads to formation of the active form, N-GSDMD (He et al., 2015; Kayagaki et al., 2015; Shi et al., 2015).

Interestingly, there is evidence that pyroptosis can also occur in the absence of Casp1, and even in the absence of GSDMD (Schneider et al., 2017). In fact, some studies found that Casp3, a pro-apoptotic caspase can induce pyroptosis (Rogers et al., 2017; Wang et al., 2017). More typically, the death receptor-mediated pathway of apoptosis activates Casp3 *via* activation of Casp8 or the mitochondrial apoptotic pathway activates Casp3 *via* Casp9 (Li and Yuan, 2008). During the mediation of pyroptosis by the extrinsic or intrinsic pathways of apoptosis, GSDME functions as the effector. Several anti-cancer agents promote the cleavage of GSDME into N-GSDME, thereby promoting the death of cancer cells (Rogers et al., 2017; Wang et al., 2017; Zhou et al., 2018). N-GSDMD and N-GSDME function as executioners because they promote the formation of pores on the plasma membrane.

IL-1β and IL-18 (pro-inflammatory cytokines) are processed from precursors into biologically active forms. In contrast to other cytokines, cytokines in the IL-1 family are not released *via* typical protein secretion pathways, and the mechanisms underlying the release of IL-1β and IL-18 were only recently determined. In particular, recent studies found that several GSDMs (GSDMD, GSDMA3, and GSDME) undergo proteolytic activation of their N-terminal domains and then form large transmembrane pores (Aglietti et al., 2016; Chen et al., 2016; Ding et al., 2016; Liu et al., 2016; Wang et al., 2017). Cryo-electron microscopic analysis of the structure of the mouse GSDMA3 pore provided important insights regarding the mechanism of this pore formation (Ruan et al., 2018). In particular, the pore consists of a 27-fold symmetric, ∼0.8-MDa oligomeric assembly and has an inner diameter of 18 nm (Ruan et al., 2018). This pore allows the transfer of many soluble cytosolic contents to the extracellular space including pro-inflammatory cytokines IL-1β and IL-18 (Heilig et al., 2018; Xia et al., 2021a). However, lactate dehydrogenase (LDH), a ∼140 kDa protein whose release is often used as a marker of complete membrane rupture and lytic cell death, is too large to pass through this pore (Heilig et al., 2018)^,^ (Tinton et al., 1993).

## GSDMs and GSDME

The GSDM family is so named because these proteins have high expression along the gastrointestinal tract and dermis (Saeki et al., 2000; Tamura et al., 2007). This protein family includes GSDMA, GSDMB, GSDMC, GSDMD, GSDME (DFNA5), and DFNB59 (Pejvakin) in humans (corresponding to GSDMA1-3, GSDMC1-4, GSDMD, GSDME, and DFNB59 in mice) (Nagarajan et al., 2019). These proteins have 45% sequence homology, and most of them have similar functional domains (Feng et al., 2018). Except for DFNB59, each protein has a N-terminal domain that is responsible for pore formation domain, a C-terminal domain that functions as a regulator, and a flexible “hinge” region that joints these two domains (Op de Beeck et al., 2011). A recent study reported that the N-GSDM domain of GSDMA, GSDMD, and GSDME executed cell death by pore formation (Ding et al., 2016), and that the C-terminal domain (C-GSDM) of the full-length protein inhibited this action. Although GSDMD was the first identified and most studied member of this family, recent studies of the pyroptosis of tumors have focused on GSDME (Feng et al., 2018; Nagarajan et al., 2019).

A 1998 study first identified the GSDME gene (also called deafness autosomal dominant 5, DFNA5) on chromosome 7p15.3. Patients with specific genetic variants have autosomal dominant, progressive, sensorineural, and non-syndromic hearing loss (Van Laer et al., 1998). The wildtype GSDME has 10 exons, encodes a protein with 496 amino acids, and has a molecular weight of ∼55 kDa. Most GSDME mutations affect the inhibitory C-terminal domain, thus leading to spontaneous formation of pores and pyroptosis (Gregan et al., 2003). Although families with GSDME-mediated hearing loss have different mutations at the DNA level, they all have a deletion of exon eight and protein truncation (Yu et al., 2003; BISCHOFF et al., 2004; Cheng et al., 2007; Park et al., 2010; De Beeck et al., 2012; Chai et al., 2014; Nishio et al., 2014; Li-Yang et al., 2015; Nadol et al., 2015; Wang et al., 2018a; Booth et al., 2018) (Figure 1B).

GSDME expression varies among cells and tissues. In humans, GSDME is normally expressed in the placenta, heart, brain, and kidneys; however, in mice GSDME is expressed in the cochlea, thymus, colon, lung, brain, spleen, and small intestine (Van Laer et al., 1998; Wu et al., 2009), but not in the breasts, gastric system, or colorectal cancer due to hypermethylation of its promotor (Akino et al., 2007; Kim et al., 2008; Stoll et al., 2017). Recent studies found that chemotherapy drugs activated the Casp3/GSDME-dependent pyroptosis pathway *via* cleavage of GSDME to N-GSDME and C-GSDME, and that this led to pore formation in the membranes and pyroptosis (Rogers et al., 2017; Wang et al., 2017). Rogers et al. demonstrated that the human and mouse DFNA5 protein contained a putative Casp3 recognition motif at residues 267–270 (267DMPD270) (Rogers et al., 2017), whereas Wang et al. identified a Casp3-cleavable site at 267DMPD270 in the human protein and at 267DMLD270 in the mouse protein (Wang et al., 2017). The potential cause of these different findings may be the different methods used for identification of this motif. For example, Rogers et al. identified the Casp3 recognition site in GSDME by searching for a consensus Casp3 recognition motif in the linker region between the N-GSDME and C-GSDME domains, and then they confirmed the Casp3-cleavable site by substituting Asp270 with Glu270 in the human protein using site-directed mutagenesis (Rogers et al., 2017). In contrast, Wang et al. identified the Casp3 recognition site by using self-made Casp3 protein to cleave GSDME that was used for liposome-binding and leakage assays (Wang et al., 2017).

Interestingly, GSDME can act downstream and upstream of Casp3. In particular, generation of the N-GSDME by Casp3 leads to permeabilization of the mitochondrial membrane, the release of Cyto C, and the inducion of Casp3-mediated apoptosis (Tait and Green, 2010; Rogers et al., 2019). This cross-talk of the endogenous and exogenous pathways of apoptosis also promotes the activation of Casp3, thus forming a positive feedback loop (Jiang et al., 2020). GSDME cleavage into N-GSDME leads to the formation of large pores in the plasma membrane and pyroptosis, and N-GSDME also permeabilizes the mitochondrial membrane and this activates apoptosis *via* the mitochondrial pathway. Although Casp3 has pro-apoptotic effects, GSDME may be considered a “switch” that determines whether a cell undergoes pyroptosis or apoptosis; in that its level determines the type of PCD that occurs in cells with activated Casp3. In particular, Wang et al. reported that the expression of GSDME determined whether cells underwent apoptosis or pyroptosis, with a high level of GSDME leading to pyroptosis and a low level leading to apoptosis (as in patients receiving chemotherapy). In the presence of GSDME, chemotherapy drug-induced Casp3 activation often leads to pyroptosis (Rogers et al., 2017; Wang et al., 2017), because pyroptosis is a more rapid process than apoptosis (Shi et al., 2015).

However, several studies have challenged the interpretation that GSDME drives pyroptosis upon stimulation of apoptosis. For example, GSDME-deficient macrophages still underwent plasma membrane damage in response to Casp3 activators (Lee et al., 2018). Another study found that during mitochondrial apoptosis, GSDME was not required for channel formation, and that it had a redundant role in the lysis of macrophage that was downstream of the ripoptosome (Chen et al., 2019). In agreement, Tixeira et al. reported that GSDME was unnecessary for the pyroptosis-mediated regulation of human T cells and monocytes (Tixeira et al., 2018) and Lee et al. demonstrated that GSDME was not required for pyroptosis in caspase-1^−/−^caspase-11^−/−^ bone morrow-derived macrophages treated with flagellin, cytochrome, or the Fas ligand (Lee et al., 2018). Taking together, these findings suggest that GSDME-mediated pyroptosis occurs only in cells that have specific internal conditions or only in specific types of cells.

### Pyroptosis Mediates Different Types of Renal Cell Damage in DN

The increasing prevalence of diabetes, and DN indicates the need for an improved understanding of the mechanisms underlying these diseases. DN is a complex process involving multiple factors. Excessive accumulation of glucose and lipids are the initial inducers of DN. The consequent induction of inflammation, fibrosis, hemodynamic alterations, oxidative stress, and apoptosis are the main pathogenic characteristics of DN (Barrera-Chimal and Jaisser, 2020; Kushwaha et al., 2020; Opazo-Ríos et al., 2020; Thongnak et al., 2020), but there is emerging evidence that additional signaling molecules function in the pathogenesis of DN. These include renal endothelial-related molecules (endothelial sirtuin 3 [SIRT3] (Wang et al., 2019c), endothelial glucocorticoid receptors [E-GRs] (Srivastava et al., 2021a), and endothelial fibroblast growth factor receptor 1 [FGFR1]) (Typiak et al., 2021), and the podocyte-glucocorticoid receptor (P-GR) (Srivastava et al., 2021b). E-GR and P-GR have anti-fibrotic effects, and their loss or knock-out-promotes fibrosis and accelerates DN (Srivastava et al., 2021a; Srivastava et al., 2021b).

However, FGFR1, together with an appropriate rate of glycolysis in podocytes, is essential for maintenance of the normal filtration barrier (Typiak et al., 2021). The endothelial protein SIRT3 regulates the redox balance and this decreases hyperglycemia-induced vascular inflammation, increases cell survival, and restores AMPK-mediated mitochondrial homeostasis (Wang et al., 2019c). Recent research identified numerous drugs that potentially provide nephroprotection in DN, including a DPP-4 inhibitor (linagliptin) (Kanasaki et al., 2014), a sodium-glucose cotransporter two inhibitor (empagliflozin) (Scheen, 2015), glycolysis inhibitors (Zeng et al., 2020), ACE inhibitors (Kelleher, 1990), ARBs (Tu et al., 2015), peptide AcSDKP (Nitta et al., 2016) and SIRT3 activators (Wang et al., 2019c). The application of these drugs has achieved good results, but there is still a long way for the prevention and treatment of DN. Recently, it is reported that pyroptosis promotes the progression of diabetes and several diabetic complications including DN (Wang, et al., 2019a; An, et al., 2020; Gu et al., 2019; Li, et al., 2020a; Cheng, et al., 2021; Chen, et al., 2020; Han, et al., 2021; Li, et al., 2021a; Li, et al., 2021b; Zuo, et al., 2021). Thus, drugs targeting different pyroptotic pathways have potential as therapies for treatment of diabetes and its complications. Below, we concisely expound the possible mechanisms of pyroptosis mediating the various cell damages in DN.

#### Pyroptosis Mediates Damage of Endothelial Cells in DN

Previous studies (Shahzad et al., 2015) identified the activation of inflammasomes in endothelial cells and podocytes *in vivo* (humans and mice with diabetes), and *in vitro* (glucose-stressed glomerular endothelial cells and podocytes). Blocking the expression of NLRP3 or Casp1 in bone marrow-derived cells did not prevent the development of DN in mice; however, mice that were deficient in NLRP3 were protected from DN even when receiving transplantation of wild-type bone marrow. An *in vitro* study of glomerular endothelial cells (GECs) reported that addition of a high dose of glucose (HG) promoted pyroptosis, but this was inhibited by silencing of GSDMD, thus indicating the participation of pyroptosis in the injury of these cells (Gu et al., 2019).

#### Pyroptosis Induces Podocyte Injury in DN

Podocytes are important for the proper function of the glomerular filtration barrier, and their injury or loss can lead to proteinuria. Cheng et al. (2021) studied mice with DN and showed that the increased expression of Casp11 and the increased cleavage of GSDMD in podocytes, correlated with reduced expression of two podocyte markers (nephrin and podocin), and with the loss and fusion of podocyte foot processes. Knockout of Casp11 or GSDMD mitigated these changes in DN mice model. Their studies of human podocytes (Cheng et al., 2021) treated with HG led to similar results. Specifically, knockdown of Casp4 (or GSDMD) with siRNA significantly reduced the levels of Casp4 (or N-GSDMD), and also reduced the levels of NF-κB, IL-1β, and IL-18, nephrin, and podocin. Zhang et al. Gao et al. (2014, 2015)) found that HG-induced activation of NADPH oxidase was driven by thioredoxin interacting protein (TXNIP), and that this increased NLRP3 inflammasome activation in podocytes and ultimately led to podocyte injury. Thus, blockage of this signaling may have promise as a treatment for DN. Yang et al. Liu et al. (2018)) demonstrated that TLR4 knockdown attenuated HG-induced podocyte injury by targeting the NALP3/ASC/Casp1 signaling pathway.

#### Pyroptosis Causes Renal Tubular Epithelial Cell Injury in DN

The renal damage induced by pyroptosis in DN is not limited to GECs and podocytes. In fact, GSDMD is a critical protein in pyroptosis, its role in regulating renal tubular injury in DN was widely discussed. As the executioner of pyroptosis, GSDMD functions in various pathways that contributes to this injury. TLR-4/NF-kappaβ signaling is a common pathway in inflammation. Wang et al. (2019a), reported that inhibition of NF-κB decreased the levels of Cas1, N-GSDMD, and secretion of IL-18 and IL-1β. Ke et al. (2020) studied a rat model of DN and reported that activation of the TXNIP/NLRP3 axis led to induction of inflammation and pyroptosis, and silencing of TXNIP inhibited inflammation and pyroptosis, as well as renal damage. They performed *in vitro* to examine pyroptosis-mediated damage of NRK-52E cells (Ke et al., 2020). microRNA miR-200a, which is degraded by an endoplasmic reticulum stress (ERS)-related factor (IRE1α), binds to TXNIP. Thus, inhibiting IRE1α increased the level of miR-200a, and decreased the level of TXNIP. Conversely, inhibiting miR-200a in HG-induced NRK-52E cells partially blocked pyroptosis that was mediated by STF-083010, an IRE1α RNase specific inhibito. Li et al. (2017), investigated the role of long noncoding RNA MALAT1 and its downstream molecules in the pyroptosis-induced injury of HK-2 cells. Their data demonstrated that HG-treated HK-2 cells had an increased level of MALAT1 and a decreased level of miR-23c, and this led to NLRP3 activation and initiation of pyroptosis.

Sun and his team (Han et al., 2018) found an increased level of mtROS-TXNIP-NLRP3 inflammasomes in the kidneys of patients with DN and in diabetic (*db*/*db*) mice. Addition of MitoQ to HK-2 cells prevented the dissociation of TRX from TXNIP and blocked the interaction of TXNIP and NLRP3, and this inhibited the activity of NLRP3 inflammasomes and the maturation of IL-1β. Pretreatment of these cells with *TXNIP* siRNA increased the effect of MitoQ, and pretreatment with monosodium urate (MSU) and *TRX* siRNA blocked this effect. This suggests that activation of mitochondrial ROS-TXNIP/NLRP3/IL-1β signaling leads to tubular oxidative injury, and that this can be blocked by MitoQ due to its inhibition of mtROS production (Han et al., 2018).

In addition, long noncoding RNAs (lncRNAs) also reduced the severity of DN *via* their regulation of miRNAs in HK-2 cells. In particular, Xie et al. (2019) found that HG-treated HK-2 cells had decreased expression of growth arrest specific 5 (lncRNA *GAS5*), and that *GAS5* overexpression downregulated several proteins related to pyroptosis (GSDMD-N, Casp1, NLRP3, and IL-1β). Notably, interference by *miR-452-5p* led to the same changes as overexpression of *GAS5* in these same cells, and inhibition of *GAS5* reversed the effect of *miR-452-5p* interference. This led to the conclusion that the lncRNA *GAS5*/*miR-452-5p* axis regulated HG-induced pyroptosis in renal tubular cells. In addition, studies of the lncRNA *KCNQ1OT1*/*miR-506-3p* pathway have provided additional insights into the pathogenesis and possible treatment of DN. In particular, downregulation of *KCNQ1OT1* in HG-treated HK-2 cells led to upregulation of *miR-506-3p*, and this was followed by reduced pyroptosis, inflammation, and oxidative stress (Zhu et al., 2020).

#### Pyroptosis Leads to Glomerular Mesangial Cell Injury in DN

Other studies examined the role of pyrolysis of glomerular mesangial cells in DN. For example, Zhan et al. (2020) established a rat model of DN and found that mesangial cell pyroptosis occurred during the pathogenesis of DN, and that upregulation of the lncRNA nuclear paraspeckle assembly transcript 1 (*NEAT1*) increased the levels of pyroptosis-associated proteins. They also examined the interactions of *NEAT1* with pyroptosis using an *in vitro* DN model (HG-treated HBZY1 cells). Their *in vitro* results indicated that *NEAT1* altered the *miR-34c/*NLRP3 axis and thereby regulated pyroptosis in these cells.

### GSDME Mediated Pyroptosis Presents Great Potential Targeting DN

A 2017 study first reported that chemotherapy drugs specifically cleaved GSDME, and that this generated N-GSDME, pore formation in plasma membranes, and pyroptosis (Rogers et al., 2017). Casp3 is thus a pro-apoptotic caspase and is also responsible for GSDME cleavage, and GSDME status determines whether a cell undergoes pyroptosis or apoptosis. In agreement with this interpretation, pyroptosis occurs in normal human primary cells that express GSDME (epidermal keratinocytes, placental epithelial cells, and umbilical artery smooth muscle cells) and in cancer cells (neuroblastoma, skin melanoma, and gastric cancer cells) (Wang et al., 2017; Wang et al., 2018b; Zhang et al., 2019). However, cells that do not express GSDME undergo apoptosis. For example, mice that lack GSDME (GSDME^−/-^) were protected from tissue injury due to chemotherapy (Wang et al., 2017). Indeed, traditional chemotherapeutic drugs, such as cisplatin, are commonly used to treat various cancers, including lung, bladder, and ovarian cancer (Pabla and Dong, 2008; BROXTERMAN et al., 2009; Ozkok and Edelstein, 2014; Gabizon et al., 2016). Chemotherapeutic drug-induced nephrotoxicity reportedly occurs in one-third of cancer patients, which limits the clinical application of these drugs (Izzedine, 2018; Volarevic et al., 2019). Chen and his team demonstrated that renal tubular epithelial cell pyroptosis induced by chemotherapy drugs (cisplatin or doxorubicin) was mediated by ROS-JNK-Casp3-GSDME signaling (Shen et al., 2021). Therefore treatments that target GSDME signaling may reduce this specific cause of drug-induced nephrotoxicity. Their research also examined the role of GSDME in renal cellular pyroptosis and in the pathogenesis of cisplatin-induced acute kidney injury (AKI) in mice that received cisplatin. The results indicated that GSDME^−/−^ mice had less severe renal injury AKI and reduced inflammation. This role of GSDME in promotion of pyroptosis also occurred in human tubular epithelial cells (TECs) *in vitro*. Interestingly, inhibition of Casp3 blocked the cleavage of GSDME to N-GSDME, and this ameliorated cisplatin-induced pyroptosis and kidney dysfunction (Xia et al., 2021b).

Caspase/GSDME-mediated pyroptosis also occurs in other inflammatory diseases. Tan et al. studied bone marrow-derived macrophages (BMDMs) that were treated with the MCC950 (a specific inhibitor of the NLRP3) and RAW264.7 cells that lcaked in ASC. Their results showed that cell lysis induced by extracellular ATP had histological similarities to the canonical pyroptosis in these cells, but Casp1 was not activated, and there was no cleavage of GSDMD. Instead, this activated Casp8/9 (apoptotic initiator) and Casp3/7 (apoptotic executioner), and this led to formation of N-GSDME and pyroptosis. Notably, a Casp3 inhibitor reduced the N-GSDME production and cell lysis that were induced by extracellular ATP were ameliorated by. When BMDMs did not receive MCC950 treatment, the addition of ATP led to the formation of ASC that co-localized with Casp8, but there was no such effect in BMDMs that received MCC950 treatment. Their sdudies of the RAW264.7 cells indicated that knockdown of GSDME by an siRNA attenuated ATP-induced cell lysis and the release of high mobility group box-1 protein (HMGB1). Collectively, the results of these studies indicate that ATP induces pyroptosis of macrophages *via* the Casp3/GSDME axis when there is blockage of the canonical NLRP3 pathway. This suggests there is an alternative mechanism for prevention of pathogen invasion (Zeng et al., 2019).

In addition to inflammatory diseases, pyroptosis also functions in fibrotic diseases. For example, research on obstructive nephropathy by Xu et al. showed that Casp3/GSDME-mediated pyroptosis occurred in renal parenchyma but not in hematopoietic cells. The studies of renal parenchyma indicated pyroptosis was related to the onset of renal tubule injury due to ureteral obstruction, and subsequently exacerbated hydronephrosis, inflammation, and fibrosis. Deletion of Casp3 or GSDME reduced renal tubule damage, inflammation, hydronephrosis, and kidney fibrosis (Li et al., 2021b).

In a study of microelement molybdenum and environmental heavy metal contaminant cadmium-induced nephrotoxicity, GSDME was confirmed to be involved in this injury (Zhang et al., 2021). In addition, it was reported that GSDMEb-mediated pyroptosis promoted LPS-induced AKI in zebrafish and renal tubule cell injury from zebrafish larvae. Gene knockout of GSDMEb can block these damages induced by LPS (Wang et al., 2020b). Many studies have examined the effect of GSDME-mediated pyroptosis in kidney diseases, and these studies used multiple interventions, in vitro models, and in vivo models. Table 1 summarized present studies exploring GSDME-mediated pyroptosis in kidney diseases.

**TABLE 1:** Summary of studies exploring GSDME-mediated pyroptosis in kidney diseases.

	Different kinds of models	Intervention	Result	Reference
*In vivo*	STZ-induced diabetic nephropathy in mice	Z-DEVD-FMK	Protective	Wen et al., 2020
	Murine UUO model	GSDME^−/−^	Protective	Li et al., 2021b
		Casp3^−/−^	Protective	Li et al., 2021b
		Ksp-Cre ×Casp3^fl/fl2^	Protective	Li et al., 2021b
		Vav-Cre × Casp3^fl/fl2^	No difference	Li et al., 2021b
		Ksp-Cre ×HMGB1fL/fl	Protective	Li et al., 2021b
		TNF-α neutralizing antibody	Protective	Li et al., 2021b
		HMGB1 neutralizing antibody	No difference	Li et al., 2021b
		HMGB1+ TNF-α neutralizing antibodies	No difference	Li et al., 2021b
	Murine R-UUO model	GSDME^−/−^	Protective	Li et al., 2021b
		Casp3^−/−^	Protective	Li et al., 2021b
		TNF-α neutralizing antibody	Protective	Li et al., 2021b
		HMGB1 neutralizing antibody	Protective	Li et al., 2021b
		HMGB1+ TNF-α neutralizing antibodies	Protective	Li et al., 2021b
	Murine cisplatin-induced AKI model	GSDME^−/−^	Protective	Xia et al., 2021b
		pEGFP-N1-GSDME-N plasmids	Destructive	Xia et al., 2021b
		Z-DEVD-FMK	Protective	Xia et al., 2021b
		Ac-DMLD-CMK	Protective	Shen et al., 2021
	Murine renal IR injury model	GSDME^−/−^	Protective	Xia et al., 2021b
	Molybdenum and cadmium co-induced nephrotoxicity in ducks	No introduce	No introduce	Zhang et al., 2021
	LPS-induced septic AKI in zebrafish	Caspy2^−/−^	Protective	Wang et al., 2020b
		GSDMEb^−/−^	Protective	Wang et al., 2020b
		Caspy^−/−^	No difference	Wang et al., 2020b
		Pycard^−/−^	No difference	Wang et al., 2020b
	LPS-induced septic AKI in zebrafish	Casp3a^−/−^	No difference	Wang et al., 2020b
		GSDMEa^−/−^	No difference	Wang et al., 2020b
		Ac-FEID-CMK	Protective	Wang et al., 2020b
	LPS-induced septic AKI in mice^6^	Casp11^−/−^	Protective	Wang et al., 2020b
		GSDMD^−/−^	Protective	Wang et al., 2020b
*In vitro*	High-glucose treatment induced HK-2 injury	Z-DEVD-FMK	Protective	Wen et al., 2020
		GSDME inhibition by shRNA	Protective	Wen et al., 2020
	TNFα induced RTCs injury under the condition of OGSD	GSDME-expressing lentivirus	Destructive	Li et al., 2021b
		GSDME^−/−^	Protective	Li et al., 2021b
		Casp3^−/−^	Protective	Li et al., 2021b
		HMGB1^Ksp−/−^	Protective	Li et al., 2021b
	Cisplatin induced HK-2 injury	plasmids with overexpressed GSDME-N	Destructive	Li et al., 2021b
		plasmids with overexpressed FL-GSDME	Destructive	Li et al., 2021b
		siRNA of Casp3	Protective	Shen et al., 2021, Xia et al., 2021b
		siRNA of Casp7	Destructive	Shen et al., 2021
		siRNA of Casp9	No difference	Shen et al., 2021
		siRNA of FL-GSDME	Protective	Xia et al., 2021b
		Z-DEVD-FMK	Protective	Shen et al., 2021
		GSK′872	No difference	Shen et al., 2021
		GSDME^−/−^	Protective	Shen et al., 2021
	Doxorubicin-induced pyroptosis in cultured HK-2 cells	Z-DEVD-FMK	Protective	Shen et al., 2021
		siRNA of Casp3	Protective	Shen et al., 2021
		siRNA of Casp7	Destructive	Shen et al., 2021
		siRNA of Casp9	No difference	Shen et al., 2021
		GSDME^−/−^	Protective	Shen et al., 2021
	Doxorubicin-induced pyroptosis in human podocytes	siRNA of Casp3	Protective	Shen et al., 2021
		siRNA of GSDME	Protectvie	Shen et al., 2021
	LPS-induced injury in renal tubule cell of zebrafish larvae	Caspy2^−/−^	Protective	Wang et al., 2020b
		GSDMEb^−/−^	Protective	Wang et al., 2020b

STZ, streptozotocin; UUO, unilateral ureteral obstruction; Casp3, caspase-3; HMGB1, high mobility group box-1, protein; TNF-a, tumor necrosis factor α; R-UUO, reversible unilateral ureteral obstruction; AKI, acute kidney injury; IR, ischemia-reperfusion; LPS, lipopolysaccharide; Casp11, caspase-11; HK-2, human kidney tubule epithelial cells; RTCs, renal primary tubular cells; shRNA, short hairpin RNA; OGSD, oxygen-glucose-serum deprivation; GSDME-N, N-terminal domain of GSDME; FL-GSDME, full length of GSDME; Casp7, caspase-7; Casp9, caspase-9; siRNA, small interfering RNA.

**FIGURE 2:**
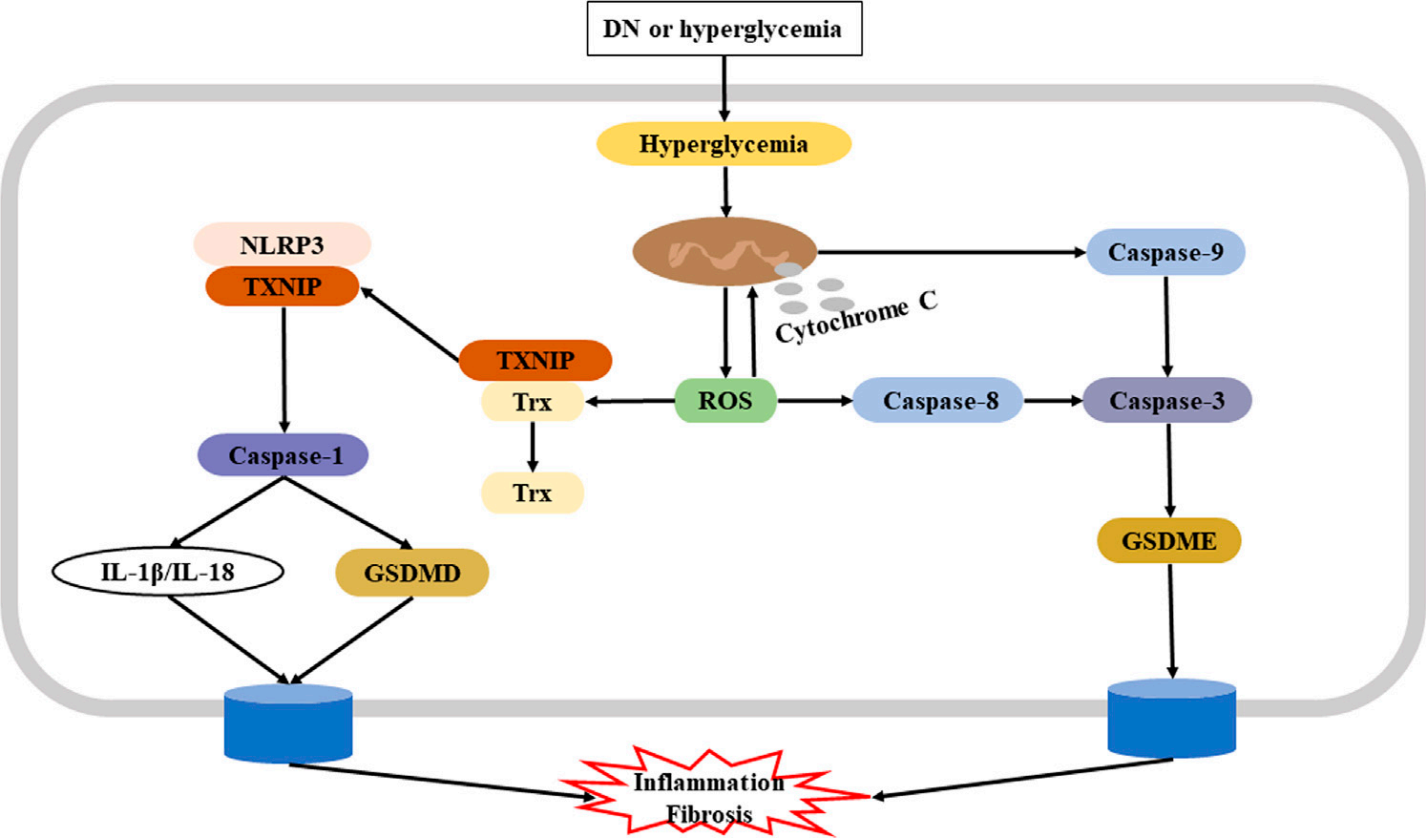
Potential mechanism of pyroptosis in the pathogenesis of DN. Hyperglycemia-induced production of ROS plays a key role in the initiation and progression of DN. **Left:** One ROS signaling pathway leads to dissociation of TXNIP from Trx and subsequent activation of NLRP3 and the Casp1/GSDMD canonical pyroptosis pathway. **Right:** Another ROS signaling pathway leads to activation of Casp3 *via* Casp8 or via mitochondrial dysfunction; in both cases, there is induction of Casp3/GSDME-mediated pyroptosi, leading to inflammation and fibrosis. **Abbreviation:** DN, diabetic nephropathy; ROS, reactive oxygen species; TXNIP, thioredoxin interacting protein; Trx, thioredoxin; NLRP3, NOD-like receptor family pyrin domain containing three; Casp1, caspase-1; Casp3, caspase-3; GSDME, gasdermin E.

Although there are a few reports about the role of GSDME-dependent pyroptosis in DN, its specific roles are uncertain. A recent study confirmed the underlying mechanism of renoprotection provided by GSDME regulation in human tubular cells. In particular, Wen et al. showed that the Casp3 inhibitor Z-DEVD-FMK reduced albuminuria, improved renal function, and blocked tubulointerstitial fibrosis in mice with diabetes. It is likely that these nephroprotective effects were due to its inhibition of GSDME. Studies of HG-treated HK-2 cells reported molecular and histological features of secondary necrosis, with evidence of GSDME cleavage, swelling of the cells, and release of cellular contents (Wen et al., 2020). In addition to fibrosis, inflammation also contributes to the progression of DN (Wada and Makino, 2016). Renal inflammation and fibrosis are crucial in promoting the onset and progression of DN, and the findings presented above demonstrate that Casp3/GSDME-triggered pyroptosis contributed to the inflammation and fibrosis that are characteristic of AKI and CKD. Figure 2 shows the potential mechanism of pyroptosis in the pathogenesis of DN.

Therefore, we speculate that experimental interventions that target Casp3/GSDME dependent pyroptosis will provide novel insights for the development of new treatments for DN. There are currently several GSDME-derived Casp3 inhibitors that differ from previously described classical Casp3 inhibitors (e.g., Z-DEVD-FMK). For example, the human/mouse GSDME cleavage site peptide DMPD/DMLD binds to the catalytic domain of Casp3. In addition, the derived inhibitor Ac-DMPD/DMLD-CMK significantly suppressed Casp3 activation and reduced the levels of downstream effectors GSDME, thereby preventing apoptosis and pyroptosis. Initial studies of these inhibitors showed they helped to prevent *in vivo* acute hepatic failure that was induced ligation of bile duct due to their blockage of apoptosis and pyroptosis in hepatocytes and macrophages (Xu et al., 2021).

### Pharmacological Inhibitors, Future Directions and Perspective in DN

The many studies discussed above clearly indicate that Casp3/GSDME-dependent pyroptosis occurs during the onset and progression of DN. The presence of this type of pyroptosis was also confirmed in HG-treated HK-2 cells, and a selective Casp3 inhibitor (Z-DEVD-FMK) inhibited pyroptosis and fibrogenesis. However, several remaining questions must be addressed. First, because glomerular injury is responsible for albuminuria and is common during the early stages of DN (Setyaningsih et al., 2021), does Casp3/GSDME-mediated pyroptosis contribute to the injury of other renal cells in addition to damaging renal tubular epithelial cells? Second, does Casp3/GSDME-mediated pyroptosis interact with other signaling pathways, and if so, are these interactions upstream or downstream?

The selective Casp3 inhibitor Z-DEVD-FMK shows great potential as a treatment for DN. There is also evidence that the Ac-DMPD/DMLD-CMK (a GSDME-derived inhibitor) protects against acute hepatic failure by regulating apoptotic and pyroptotic events in hepatocytes and macrophages. Thus, does Ac-DMPD/DMLD-CMK also have a renoprotective effect in DN? If so, which is more effective, Z-DEVD-FMK or Ac-DMPD/DMLD-CMK? Finally, can gene knockout or interference, especially at the organ or tissue level, be an effective treatment? Although there is still a long way to go, further research of Casp3/GSDME-mediated pyroptosis may provide answers to these and other important questions and help to identify new therapies for DN.

## References

[B1] Aglietti R. A. Dueber E. C. (2017). Recent Insights into the Molecular Mechanisms Underlying Pyroptosis and Gasdermin Family Functions. Trends Immunol. 38 (4), 261–271. 10.1016/j.it.2017.01.003 28196749

[B2] Aglietti R. A. Estevez A. Gupta A. Ramirez M. G. Liu P. S. Kayagaki N. (2016). GsdmD P30 Elicited by Caspase-11 during Pyroptosis Forms Pores in Membranes. Proc. Natl. Acad. Sci. U S A. 113 (28), 7858PMC4948338–63. 10.1073/pnas.1607769113 27339137 PMC4948338

[B3] Akino K. Toyota M. Suzuki H. Imai T. Maruyama R. Kusano M. (2007). Identification of DFNA5 as a Target of Epigenetic Inactivation in Gastric Cancer. Cancer Sci. 98 (1), 88–95. 10.1111/j.1349-7006.2006.00351.x 17083569 PMC11158324

[B4] An X. Zhang Y. Cao Y. Chen J. Qin H. Yang L. (2020). Punicalagin Protects Diabetic Nephropathy by Inhibiting Pyroptosis Based on TXNIP/NLRP3 Pathway. Nutrients 12 (5), 1516. 10.3390/nu12051516 PMC7284711 32456088

[B5] Argüelles S. Guerrero-Castilla A. Cano M. Muñoz M. F. Ayala A. (2019). Advantages and Disadvantages of Apoptosis in the Aging Process. Ann. N. Y Acad. Sci. 1443 (1), 20–33. 10.1111/nyas.14020 30839127

[B6] Barrera-Chimal J. Jaisser F. (2020). Pathophysiologic Mechanisms in Diabetic Kidney Disease: A Focus on Current and Future Therapeutic Targets. Diabetes Obes. Metab. 22 Suppl 1 (Suppl. 1), 16–31. 10.1111/dom.13969 32267077

[B7] Bedoui S. Herold M. J. Strasser A. (2020). Emerging Connectivity of Programmed Cell Death Pathways and its Physiological Implications. Nat. Rev. Mol. Cel Biol 21 (11), 678–695. 10.1038/s41580-020-0270-8 32873928

[B8] Belavgeni A. Meyer C. Stumpf J. Hugo C. Linkermann A. (2020). Ferroptosis and Necroptosis in the Kidney. Cell Chem Biol 27 (4), 448–462. 10.1016/j.chembiol.2020.03.016 32302582

[B9] Bischoff A. M. Luijendijk M. W. Huygen P. L. van Duijnhoven G. De Leenheer E. M. Oudesluijs G. G. (2004). A Novel Mutation Identified in the DFNA5 Gene in a Dutch Family: a Clinical and Genetic Evaluation. Audiol. Neurootol. 9 (1), 34–46. 10.1159/000074185 14676472

[B10] Boise L. H. Collins C. M. (2001). Salmonella-induced Cell Death: Apoptosis, Necrosis or Programmed Cell Death? Trends Microbiol. 9 (2), 64–67. 10.1016/s0966-842x(00)01937-5 11173244

[B11] Booth K. T. Azaiez H. Kahrizi K. Wang D. Zhang Y. Frees K. (2018). Exonic Mutations and Exon Skipping: Lessons Learned from DFNA5. Hum. Mutat. 39 (3), 433–440. 10.1002/humu.23384 29266521 PMC5805621

[B12] Brault M. Oberst A. (2017). Controlled Detonation: Evolution of Necroptosis in Pathogen Defense. Immunol. Cel Biol 95 (2), 131–136. 10.1038/icb.2016.117 PMC6855669 27909314

[B13] Broxterman H. J. Gotink K. J. Verheul H. M. W. (2009). Understanding the Causes of Multidrug Resistance in Cancer: a Comparison of Doxorubicin and Sunitinib [J]. Drug Resist. Updat 12 (4-5), 114–126. 10.1016/j.drup.2009.07.001 19648052

[B14] Chai Y. Chen D. Wang X. Wu H. Yang T. (2014). A Novel Splice Site Mutation in DFNA5 Causes Late-Onset Progressive Non-syndromic Hearing Loss in a Chinese Family. Int. J. Pediatr. Otorhinolaryngol. 78 (8), 1265–1268. 10.1016/j.ijporl.2014.05.007 24933359

[B15] Chen J. Yang Y. Lv Z. Shu A. Du Q. Wang W. (2020). Study on the Inhibitive Effect of Catalpol on Diabetic Nephropathy. Life Sci. 257, 118120. 10.1016/j.lfs.2020.118120 32693244

[B16] Chen K. W. Demarco B. Heilig R. Shkarina K. Boettcher A. Farady C. J. (2019). Extrinsic and Intrinsic Apoptosis Activate Pannexin-1 to Drive NLRP3 Inflammasome Assembly. EMBO J. 38 (10), e101638. 10.15252/embj.2019101638 30902848 PMC6517827

[B17] Chen X. He W. T. Hu L. Li J. Fang Y. Wang X. (2016). Pyroptosis Is Driven by Non-selective Gasdermin-D Pore and its Morphology Is Different from MLKL Channel-Mediated Necroptosis. Cell Res 26 (9), 1007–1020. 10.1038/cr.2016.100 27573174 PMC5034106

[B18] Cheng J. Han D. Y. Dai P. Sun H. J. Tao R. Sun Q. (2007). A Novel DFNA5 Mutation, IVS8+4 A>G, in the Splice Donor Site of Intron 8 Causes Late-Onset Non-syndromic Hearing Loss in a Chinese Family. Clin. Genet. 72 (5), 471–477. 10.1111/j.1399-0004.2007.00889.x 17868390

[B19] Cheng Q. Pan J. Zhou Z. L. Yin F. Xie H. Y. Chen P. P. (2021). Caspase-11/4 and Gasdermin D-Mediated Pyroptosis Contributes to Podocyte Injury in Mouse Diabetic Nephropathy. Acta Pharmacol. Sin 42 (6), 954–963. 10.1038/s41401-020-00525-z 32968210 PMC8149386

[B20] Choi M. E. Price D. R. Ryter S. W. Choi A. M. K. (2019). Necroptosis: a Crucial Pathogenic Mediator of Human Disease. JCI Insight 4 (15), e128834. 10.1172/jci.insight.128834 PMC6693822 31391333

[B21] Cohen G. M. (1997). Caspases: the Executioners of Apoptosis. Biochem. J. 326 (Pt 1), 1–16. 10.1042/bj3260001 9337844 PMC1218630

[B22] Conrad M. Angeli J. P. Vandenabeele P. Stockwell B. R. (2016). Regulated Necrosis: Disease Relevance and Therapeutic Opportunities. Nat. Rev. Drug Discov. 15 (5), 348–366. 10.1038/nrd.2015.6 26775689 PMC6531857

[B23] Cookson B. T. Brennan M. A. (2001). Pro-inflammatory Programmed Cell Death. Trends Microbiol. 9 (3), 113–114. 10.1016/s0966-842x(00)01936-3 11303500

[B24] De Beeck K. O. Van Laer L. Van Camp G. (2012). DFNA5, a Gene Involved in Hearing Loss and Cancer: a Review. Ann. Otol Rhinol Laryngol. 121 (3), 197–207. 10.1177/000348941212100310 22530481

[B25] Ding J. Wang K. Liu W. She Y. Sun Q. Shi J. (2016). Erratum: Pore-Forming Activity and Structural Autoinhibition of the Gasdermin Family. Nature 540 (7631), 150. 10.1038/nature20106 27706141

[B26] Durand P. M. Rashidi A. Michod R. E. (2011). How an Organism Dies Affects the Fitness of its Neighbors. Am. Nat. 177 (2), 224–232. 10.1086/657686 21460558

[B27] Elmore S. (2007). Apoptosis: a Review of Programmed Cell Death. Toxicol. Pathol. 35 (4), 495–516. 10.1080/01926230701320337 17562483 PMC2117903

[B28] Feng S. Fox D. Man S. M. (2018). Mechanisms of Gasdermin Family Members in Inflammasome Signaling and Cell Death. J. Mol. Biol. 430 (18 Pt B), 3068–3080. 10.1016/j.jmb.2018.07.002 29990470

[B29] Frank D. Vince J. E. (2019). Pyroptosis versus Necroptosis: Similarities, Differences, and Crosstalk. Cell Death Differ 26 (1), 99–114. 10.1038/s41418-018-0212-6 30341423 PMC6294779

[B30] Friedlander A. M. (1986). Macrophages Are Sensitive to Anthrax Lethal Toxin through an Acid-dependent Process. J. Biol. Chem. 261 (16), 7123–7126. 10.1016/s0021-9258(17)38364-3 3711080

[B31] Gabizon A. A. Patil Y. La-Beck N. M. (2016). New Insights and Evolving Role of Pegylated Liposomal Doxorubicin in Cancer Therapy. Drug Resist. Updat 29, 90–106. 10.1016/j.drup.2016.10.003 27912846

[B32] Galluzzi L. Kepp O. Krautwald S. Kroemer G. Linkermann A. (2014). Molecular Mechanisms of Regulated Necrosis. Semin. Cel Dev Biol 35, 24–32. 10.1016/j.semcdb.2014.02.006 24582829

[B33] Galluzzi L. Vitale I. Aaronson S. A. Abrams J. M. Adam D. Agostinis P. (2018). Molecular Mechanisms of Cell Death: Recommendations of the Nomenclature Committee on Cell Death 2018. Cel Death Differ 25 (3), 486–541. 10.1038/s41418-017-0012-4 PMC5864239 29362479

[B34] Gao P. He F. F. Tang H. Lei C. T. Chen S. Meng X. F. (2015). NADPH Oxidase-Induced NALP3 Inflammasome Activation Is Driven by Thioredoxin-Interacting Protein Which Contributes to Podocyte Injury in Hyperglycemia. J. Diabetes Res. 2015, 504761. 10.1155/2015/504761 25834832 PMC4365330

[B35] Gao P. Meng X. F. Su H. He F. F. Chen S. Tang H. (2014). Thioredoxin-interacting Protein Mediates NALP3 Inflammasome Activation in Podocytes during Diabetic Nephropathy. Biochim. Biophys. Acta 1843 (11), 2448–2460. 10.1016/j.bbamcr.2014.07.001 25017793

[B36] Green D. R. (2019). The Coming Decade of Cell Death Research: Five Riddles. Cell 177 (5), 1094–1107. 10.1016/j.cell.2019.04.024 31100266 PMC6534278

[B37] Gregan J. Van Laer L. Lieto L. D. Van Camp G. Kearsey S. E. (2003). A Yeast Model for the Study of Human DFNA5, a Gene Mutated in Nonsyndromic Hearing Impairment. Biochim. Biophys. Acta 1638 (2), 179–186. 10.1016/s0925-4439(03)00083-8 12853124

[B38] Gu J. Huang W. Zhang W. Zhao T. Gao C. Gan W. (2019). Sodium Butyrate Alleviates High-Glucose-Induced Renal Glomerular Endothelial Cells Damage via Inhibiting Pyroptosis. Int. Immunopharmacol 75, 105832. 10.1016/j.intimp.2019.105832 31473434

[B39] Hagar J. A. Powell D. A. Aachoui Y. Ernst R. K. Miao E. A. (2013). Cytoplasmic LPS Activates Caspase-11: Implications in TLR4-independent Endotoxic Shock. Science 341 (6151), 1250–1253. 10.1126/science.1240988 24031018 PMC3931427

[B40] Han J. Zuo Z. Shi X. Zhang Y. Peng Z. Xing Y. (2021). Hirudin Ameliorates Diabetic Nephropathy by Inhibiting Gsdmd-Mediated Pyroptosis. Cell Biol Toxicol. 10.1007/s10565-021-09622-z 34212273

[B41] Han Y. Xu X. Tang C. Gao P. Chen X. Xiong X. (2018). Reactive Oxygen Species Promote Tubular Injury in Diabetic Nephropathy: The Role of the Mitochondrial Ros-Txnip-Nlrp3 Biological axis. Redox Biol. 16, 32–46. 10.1016/j.redox.2018.02.013 29475133 PMC5842313

[B42] Harjutsalo V. Groop P. H. (2014). Epidemiology and Risk Factors for Diabetic Kidney Disease. Adv. Chronic Kidney Dis. 21 (3), 260–266. 10.1053/j.ackd.2014.03.009 24780453

[B43] He W. T. Wan H. Hu L. Chen P. Wang X. Huang Z. (2015). Gasdermin D Is an Executor of Pyroptosis and Required for Interleukin-1β Secretion. Cel Res 25 (12), 1285–1298. 10.1038/cr.2015.139 PMC4670995 26611636

[B44] Heilig R. Dick M. S. Sborgi L. Meunier E. Hiller S. Broz P. (2018). The Gasdermin-D Pore Acts as a Conduit for IL-1β Secretion in Mice. Eur. J. Immunol. 48 (4), 584–592. 10.1002/eji.201747404 29274245

[B45] Hersh D. Monack D. M. Smith M. R. Ghori N. Falkow S. Zychlinsky A. (1999). The Salmonella Invasin SipB Induces Macrophage Apoptosis by Binding to Caspase-1. Proc. Natl. Acad. Sci. U S A. 96 (5), 2396–2401. 10.1073/pnas.96.5.2396 10051653 PMC26795

[B46] Hilbi H. Chen Y. Thirumalai K. Zychlinsky A. (1997). The Interleukin 1beta-Converting Enzyme, Caspase 1, Is Activated during Shigella Flexneri-Induced Apoptosis in Human Monocyte-Derived Macrophages. Infect. Immun. 65 (12), 5165–5170. 10.1128/iai.65.12.5165-5170.1997 9393811 PMC175744

[B47] Huang Y. M. Xu D. Long J. Shi Y. Zhang L. Wang H. (2019). Spectrum of Chronic Kidney Disease in China: A National Study Based on Hospitalized Patients from 2010 to 2015. Nephrology (Carlton) 24 (7), 725–736. 10.1111/nep.13489 30198082

[B48] Imai H. Matsuoka M. Kumagai T. Sakamoto T. Koumura T. (2017). Lipid Peroxidation-dependent Cell Death Regulated by GPx4 and Ferroptosis. Curr. Top. Microbiol. Immunol. 403, 143–170. 10.1007/82_2016_508 28204974

[B49] Izzedine H. (2018). Drug Nephrotoxicity. Nephrol. Ther. 14 (3), 127–134. 10.1016/j.nephro.2017.06.006 29540291

[B50] Jiang M. Qi L. Li L. Li Y. (2020). The Caspase-3/GSDME Signal Pathway as a Switch between Apoptosis and Pyroptosis in Cancer. Cell Death Discov 6, 112. 10.1038/s41420-020-00349-0 33133646 PMC7595122

[B51] Jorgensen I. Miao E. A. (2015). Pyroptotic Cell Death Defends against Intracellular Pathogens. Immunol. Rev. 265 (1), 130–142. 10.1111/imr.12287 25879289 PMC4400865

[B52] Jorgensen I. Rayamajhi M. Miao E. A. (2017). Programmed Cell Death as a Defence against Infection. Nat. Rev. Immunol. 17 (3), 151–164. 10.1038/nri.2016.147 28138137 PMC5328506

[B53] Kaczanowski S. (2016). Apoptosis: its Origin, History, Maintenance and the Medical Implications for Cancer and Aging. Phys. Biol. 13 (3), 031001. 10.1088/1478-3975/13/3/031001 27172135

[B54] Kambara H. Liu F. Zhang X. Liu P. Bajrami B. Teng Y. (2018). Gasdermin D Exerts Anti-inflammatory Effects by Promoting Neutrophil Death. Cell Rep 22 (11), 2924–2936. 10.1016/j.celrep.2018.02.067 29539421 PMC5878047

[B55] Kanasaki K. Shi S. Kanasaki M. He J. Nagai T. Nakamura Y. (2014). Linagliptin-mediated DPP-4 Inhibition Ameliorates Kidney Fibrosis in Streptozotocin-Induced Diabetic Mice by Inhibiting Endothelial-To-Mesenchymal Transition in a Therapeutic Regimen. Diabetes 63 (6), 2120–2131. 10.2337/db13-1029 24574044

[B56] Kayagaki N. Stowe I. B. Lee B. L. O'Rourke K. Anderson K. Warming S. (2015). Caspase-11 Cleaves Gasdermin D for Non-canonical Inflammasome Signalling. Nature 526 (7575), 666–671. 10.1038/nature15541 26375259

[B57] Kayagaki N. Warming S. Lamkanfi M. Vande Walle L. Louie S. Dong J. (2011). Non-canonical Inflammasome Activation Targets Caspase-11. Nature 479 (7371), 117–121. 10.1038/nature10558 22002608

[B58] Kayagaki N. Wong M. T. Stowe I. B. Ramani S. R. Gonzalez L. C. Akashi-Takamura S. (2013). Noncanonical Inflammasome Activation by Intracellular LPS Independent of TLR4. Science 341 (6151), 1246–1249. 10.1126/science.1240248 23887873

[B59] Ke R. Wang Y. Hong S. Xiao L. (2020). Endoplasmic Reticulum Stress Related Factor IRE1α Regulates TXNIP/NLRP3-mediated Pyroptosis in Diabetic Nephropathy. Exp. Cel Res 396 (2), 112293. 10.1016/j.yexcr.2020.112293 32950473

[B60] Kelleher C. C. (1990). ACE Inhibitors in the Prevention and Therapy of Diabetic Nephropathy. What Is Their Role? DrugsPMID 39 (5), 639–645. 10.2165/00003495-199039050-00001 2191846

[B61] Kerr J. F. Wyllie A. H. Currie A. R. (1972). Apoptosis: a Basic Biological Phenomenon with Wide-Ranging Implications in Tissue Kinetics. Br. J. Cancer 26 (4), 239–257. 10.1038/bjc.1972.33 4561027 PMC2008650

[B62] Ketelut-Carneiro N. Fitzgerald K. A. (2020). Inflammasomes. Curr. Biol. 30 (12), R689–R694. 10.1016/j.cub.2020.04.065 32574626

[B63] Kim M. S. Chang X. Yamashita K. Nagpal J. K. Baek J. H. Wu G. (2008). Aberrant Promoter Methylation and Tumor Suppressive Activity of the DFNA5 Gene in Colorectal Carcinoma. Oncogene 27 (25), 3624–3634. 10.1038/sj.onc.1211021 18223688

[B64] Kiraz Y. Adan A. Kartal Yandim M. Baran Y. (2016). Major Apoptotic Mechanisms and Genes Involved in Apoptosis. Tumour Biol. 37 (7), 8471–8486. 10.1007/s13277-016-5035-9 27059734

[B65] Kovacs S. B. Miao E. A. (2017). Gasdermins: Effectors of Pyroptosis. Trends Cel Biol 27 (9), 673–684. 10.1016/j.tcb.2017.05.005 PMC5565696 28619472

[B66] Kuang F. Liu J. Tang D. Kang R. (2020). Oxidative Damage and Antioxidant Defense in Ferroptosis. Front Cel Dev Biol 8, 586578. 10.3389/fcell.2020.586578 PMC7527737 33043019

[B67] Kushwaha K. Sharma S. Gupta J. (2020). Metabolic Memory and Diabetic Nephropathy: Beneficial Effects of Natural Epigenetic Modifiers. Biochimie 170, 140–151. 10.1016/j.biochi.2020.01.007 31954720

[B68] Lee B. L. Mirrashidi K. M. Stowe I. B. Kummerfeld S. K. Watanabe C. Haley B. (2018). ASC- and Caspase-8-dependent Apoptotic Pathway Diverges from the NLRC4 Inflammasome in Macrophages. Sci. Rep. 8 (1), 3788. 10.1038/s41598-018-21998-3 29491424 PMC5830643

[B69] Li F. Chen Y. Li Y. Huang M. Zhao W. (2020). Geniposide Alleviates Diabetic Nephropathy of Mice through AMPK/SIRT1/NF-κB Pathway. Eur. J. Pharmacol. 886, 886173449. 10.1016/j.ejphar.2020.173449 32758570

[B70] Li H. Zhao K. Li Y. (2021). Gasdermin D Protects Mouse Podocytes against High-Glucose-Induced Inflammation and Apoptosis via the C-Jun N-Terminal Kinase (JNK) Pathway. Med. Sci. Monit. 27, e928411. 10.12659/MSM.928411 33690262 PMC7955578

[B71] Li J. Cao F. Yin H. L. Huang Z. J. Lin Z. T. Mao N. (2020). Ferroptosis: Past, Present and Future. Cell Death Dis 11 (2), 88. 10.1038/s41419-020-2298-2 32015325 PMC6997353

[B72] Li J. Yuan J. (2008). Caspases in Apoptosis and beyond. Oncogene 27 (48), 6194–6206. 10.1038/onc.2008.297 18931687

[B73] Li N. Zhao T. Cao Y. Zhang H. Peng L. Wang Y. (2020). Tangshen Formula Attenuates Diabetic Kidney Injury by Imparting Anti-pyroptotic Effects *via* the TXNIP-NLRP3-GSDMD Axis. Front. Pharmacol. 11, 623489. 10.3389/fphar.2020.623489 33584307 PMC7880163

[B74] Li P. Nijhawan D. Budihardjo I. Srinivasula S. M. Ahmad M. Alnemri E. S. (1997). Cytochrome C and dATP-dependent Formation of Apaf-1/caspase-9 Complex Initiates an Apoptotic Protease cascade. Cell 91 (4), 479–489. 10.1016/s0092-8674(00)80434-1 9390557

[B75] Li X. Zeng L. Cao C. Lu C. Lian W. Han J. (2017). Long Noncoding RNA MALAT1 Regulates Renal Tubular Epithelial Pyroptosis by Modulated miR-23c Targeting of ELAVL1 in Diabetic Nephropathy. Exp. Cel Res 350 (2), 327–335. 10.1016/j.yexcr.2016.12.006 27964927

[B76] Li Y. Yuan Y. Huang Z. X. Chen H. Lan R. Wang Z. (2021). GSDME-mediated Pyroptosis Promotes Inflammation and Fibrosis in Obstructive Nephropathy. Cel Death Differ 28 (8), 2333–2350. 10.1038/s41418-021-00755-6 PMC8329275 33664482

[B77] Li-Yang M. N. Shen X. F. Wei Q. J. Yao J. Lu Y. J. Cao X. (2015). IVS8+1 DelG, a Novel Splice Site Mutation Causing DFNA5 Deafness in a Chinese Family. Chin. Med. J. (Engl) 128 (18), 2510–2515. 10.4103/0366-6999.164980 26365971 PMC4725571

[B78] Liu X. Zhang Z. Ruan J. Pan Y. Magupalli V. G. Wu H. (2016). Inflammasome-activated Gasdermin D Causes Pyroptosis by Forming Membrane Pores. Nature 535 (7610), 153–158. 10.1038/nature18629 27383986 PMC5539988

[B79] Liu X. Q. Jiang L. Lei L. Nie Z. Y. Zhu W. Wang S. (2020). Carnosine Alleviates Diabetic Nephropathy by Targeting GNMT, a Key Enzyme Mediating Renal Inflammation and Fibrosis. Clin. Sci. (Lond) 134 (23), 3175–3193. 10.1042/CS20201207 33241846 PMC7726623

[B80] Liu Y. Xu Z. Ma F. Jia Y. Wang G. (2018). Knockdown of TLR4 Attenuates High Glucose-Induced Podocyte Injury via the NALP3/ASC/Caspase-1 Signaling Pathway. Biomed. Pharmacother. 107, 1393–1401. 10.1016/j.biopha.2018.08.134 30257355

[B81] Martinon F. Burns K. Tschopp J. (2002). The Inflammasome: a Molecular Platform Triggering Activation of Inflammatory Caspases and Processing of proIL-Beta. Mol. Cel 10 (2), 417–426. 10.1016/s1097-2765(02)00599-3 12191486

[B82] Martirosyan A. Gorvel J. P. (2013). Brucella Evasion of Adaptive Immunity. Future Microbiol. 8 (2), 147–154. 10.2217/fmb.12.140 23374122

[B83] Muzio M. Chinnaiyan A. M. Kischkel F. C. O'Rourke K. Shevchenko A. Ni J. (1996). FLICE, a Novel FADD-Homologous ICE/CED-3-like Protease, Is Recruited to the CD95 (Fas/APO-1) Death-Iinducing Signaling Complex. Cell 85 (6), 817–827. 10.1016/s0092-8674(00)81266-0 8681377

[B84] Nadol J. B. Jr Handzel O. Amr S. (2015). Histopathology of the Human Inner Ear in a Patient with Sensorineural Hearing Loss Caused by a Variant in DFNA5. Otol Neurotol 36 (10), 1616–1621. 10.1097/MAO.0000000000000888 26496673

[B85] Nagarajan K. Soundarapandian K. Thorne R. F. Li D. Li D. (2019). Activation of Pyroptotic Cell Death Pathways in Cancer: An Alternative Therapeutic Approach. Transl Oncol. 12 (7), 925–931. 10.1016/j.tranon.2019.04.010 31085408 PMC6518321

[B86] Nailwal H. Chan F. K. (2019). Necroptosis in Anti-viral Inflammation. Cel Death Differ 26 (1), 4–13. 10.1038/s41418-018-0172-x PMC6294789 30050058

[B87] Newton K. Wickliffe K. E. Dugger D. L. Maltzman A. Roose-Girma M. Dohse M. (2019). Cleavage of RIPK1 by Caspase-8 Is Crucial for Limiting Apoptosis and Necroptosis. Nature 574 (7778), 428–431. 10.1038/s41586-019-1548-x 31511692

[B88] Nishio A. Noguchi Y. Sato T. Naruse T. K. Kimura A. Takagi A. (2014). A DFNA5 Mutation Identified in Japanese Families with Autosomal Dominant Hereditary Hearing Loss. Ann. Hum. Genet. 78 (2), 83–91. 10.1111/ahg.12053 24506266

[B89] Nitta K. Shi S. Nagai T. Kanasaki M. Kitada M. Srivastava S. P. (2016). Oral Administration of N-Acetyl-Seryl-Aspartyl-Lysyl-Proline Ameliorates Kidney Disease in Both Type 1 and Type 2 Diabetic Mice via a Therapeutic Regimen. Biomed. Res. Int. 2016, 9172157. 10.1155/2016/9172157 27088094 PMC4818806

[B90] Norbury C. J. Hickson I. D. (2001). Cellular Responses to DNA Damage. Annu. Rev. Pharmacol. Toxicol. 41, 367–401. 10.1146/annurev.pharmtox.41.1.367 11264462

[B91] Op de Beeck K. Van Camp G. Thys S. Cools N. Callebaut I. Vrijens K. (2011). The DFNA5 Gene, Responsible for Hearing Loss and Involved in Cancer, Encodes a Novel Apoptosis-Inducing Protein. Eur. J. Hum. Genet. 19 (9), 965–973. 10.1038/ejhg.2011.63 21522185 PMC3179363

[B92] Opazo-Ríos L. Mas S. Marín-Royo G. Mezzano S. Gómez-Guerrero C. Moreno J. A. (2020). Lipotoxicity and Diabetic Nephropathy: Novel Mechanistic Insights and Therapeutic Opportunities. Int. J. Mol. Sci. 21 (7), 2632. 10.3390/ijms21072632 PMC7177360 32290082

[B93] Orning P. Lien E. Fitzgerald K. A. (2019). Gasdermins and Their Role in Immunity and Inflammation. J. Exp. Med. 216 (11), 2453–2465. 10.1084/jem.20190545 31548300 PMC6829603

[B94] Ozkok A. Edelstein C. L. (2014). Pathophysiology of Cisplatin-Induced Acute Kidney Injury. Biomed. Res. Int. 2014, 967826. 10.1155/2014/967826 25165721 PMC4140112

[B95] Pabla N. Dong Z. (2008). Cisplatin Nephrotoxicity: Mechanisms and Renoprotective Strategies. Kidney Int. 73 (9), 994–1007. 10.1038/sj.ki.5002786 18272962

[B96] Park H. J. Cho H. J. Baek J. I. Ben-Yosef T. Kwon T. J. Griffith A. J. (2010). Evidence for a Founder Mutation Causing DFNA5 Hearing Loss in East Asians. J. Hum. Genet. 55 (1), 59–62. 10.1038/jhg.2009.114 19911014 PMC3433838

[B97] Podgórski P. Konieczny A. Lis Ł. Witkiewicz W. Hruby Z. (2019). Glomerular Podocytes in Diabetic Renal Disease. Adv. Clin. Exp. Med. 28 (12), 1711–1715. 10.17219/acem/104534 31851794

[B98] Qiu Y. Cao Y. Cao W. Jia Y. Lu N. (2020). The Application of Ferroptosis in Diseases. Pharmacol. Res. 159, 104919. 10.1016/j.phrs.2020.104919 32464324

[B99] Rogers C. Erkes D. A. Nardone A. Aplin A. E. Fernandes-Alnemri T. Alnemri E. S. (2019). Gasdermin Pores Permeabilize Mitochondria to Augment Caspase-3 Activation during Apoptosis and Inflammasome Activation. Nat. Commun. 10 (1), 1689. 10.1038/s41467-019-09397-2 30976076 PMC6459836

[B100] Rogers C. Fernandes-Alnemri T. Mayes L. Alnemri D. Cingolani G. Alnemri E. S. (2017). Cleavage of DFNA5 by Caspase-3 during Apoptosis Mediates Progression to Secondary Necrotic/pyroptotic Cell Death. Nat. Commun. 8, 14128. 10.1038/ncomms14128 28045099 PMC5216131

[B101] Ruan J. Xia S. Liu X. Lieberman J. Wu H. (2018). Cryo-EM Structure of the Gasdermin A3 Membrane Pore. Nature 557 (7703), 62–67. 10.1038/s41586-018-0058-6 29695864 PMC6007975

[B102] Sabapathy V. Stremska M. E. Mohammad S. Corey R. L. Sharma P. R. Sharma R. (2019). Novel Immunomodulatory Cytokine Regulates Inflammation, Diabetes, and Obesity to Protect from Diabetic Nephropathy. Front. Pharmacol. 10, 572. 10.3389/fphar.2019.00572 31191312 PMC6540785

[B103] Saeedi P. Petersohn I. Salpea P. Malanda B. Karuranga S. Unwin N. (2019). Global and Regional Diabetes Prevalence Estimates for 2019 and Projections for 2030 and 2045: Results from the International Diabetes Federation Diabetes Atlas, 9th Edition. Diabetes Res. Clin. Pract. 157, 107843. 10.1016/j.diabres.2019.107843 31518657

[B104] Saeki N. Kuwahara Y. Sasaki H. Satoh H. Shiroishi T. (2000). Gasdermin (Gsdm) Localizing to Mouse Chromosome 11 Is Predominantly Expressed in Upper Gastrointestinal Tract but Significantly Suppressed in Human Gastric Cancer Cells. Mamm. Genome 11 (9), 718–724. 10.1007/s003350010138 10967128

[B105] Scheen A. J. (2015). Pharmacodynamics, Efficacy and Safety of Sodium-Glucose Co-transporter Type 2 (SGLT2) Inhibitors for the Treatment of Type 2 Diabetes Mellitus. Drugs 75 (1), 33–59. 10.1007/s40265-014-0337-y 25488697

[B106] Schneider K. S. Groß C. J. Dreier R. F. Saller B. S. Mishra R. Gorka O. (2017). The Inflammasome Drives GSDMD-independent Secondary Pyroptosis and IL-1 Release in the Absence of Caspase-1 Protease Activity. Cel Rep 21 (13), 3846–3859. 10.1016/j.celrep.2017.12.018 PMC5750195 29281832

[B107] Schroder K. Tschopp J. (2010). The Inflammasomes. Cell 140 (6), 821–832. 10.1016/j.cell.2010.01.040 20303873

[B108] Setyaningsih W. A. W. Arfian N. Fitriawan A. S. Yuniartha R. Sari D. C. R. (2021). Ethanolic Extract of *Centella asiatica* Treatment in the Early Stage of Hyperglycemia Condition Inhibits Glomerular Injury and Vascular Remodeling in Diabetic Rat Model. Evid. Based Complement. Alternat Med. 2021, 6671130. 10.1155/2021/6671130 34326888 PMC8277496

[B109] Shahzad K. Bock F. Dong W. Wang H. Kopf S. Kohli S. (2015). Nlrp3-inflammasome Activation in Non-myeloid-derived Cells Aggravates Diabetic Nephropathy. Kidney Int. 87 (1), 74–84. 10.1038/ki.2014.271 25075770 PMC4284813

[B110] Shen X. Wang H. Weng C. Jiang H. Chen J. (2021). Caspase 3/GSDME-dependent Pyroptosis Contributes to Chemotherapy Drug-Induced Nephrotoxicity. Cel Death Dis 12 (2), 186. 10.1038/s41419-021-03458-5 PMC7884686 33589596

[B111] Shi J. Gao W. Shao F. (2017). Pyroptosis: Gasdermin-Mediated Programmed Necrotic Cell Death. Trends Biochem. Sci. 42 (4), 245–254. 10.1016/j.tibs.2016.10.004 27932073

[B112] Shi J. Zhao Y. Wang K. Shi X. Wang Y. Huang H. (2015). Cleavage of GSDMD by Inflammatory Caspases Determines Pyroptotic Cell Death. Nature 526 (7575), 660–665. 10.1038/nature15514 26375003

[B113] Shi J. Zhao Y. Wang Y. Gao W. Ding J. Li P. (2014). Inflammatory Caspases Are Innate Immune Receptors for Intracellular LPS. Nature 514 (7521), 187–192. 10.1038/nature13683 25119034

[B114] Song N. Li T. (2018). Regulation of NLRP3 Inflammasome by Phosphorylation. Front. Immunol. 9, 2305. 10.3389/fimmu.2018.02305 30349539 PMC6186804

[B115] Srivastava S. P. Zhou H. Setia O. Dardik A. Fernandez-Hernando C. Goodwin J. (2021). Podocyte Glucocorticoid Receptors Are Essential for Glomerular Endothelial Cell Homeostasis in Diabetes Mellitus. J. Am. Heart Assoc. 10 (15), e019437. 10.1161/JAHA.120.019437 34308664 PMC8475689

[B116] Srivastava S. P. Zhou H. Setia O. Liu B. Kanasaki K. Koya D. (2021). Loss of Endothelial Glucocorticoid Receptor Accelerates Diabetic Nephropathy. Nat. Commun. 12 (1), 2368. 10.1038/s41467-021-22617-y 33888696 PMC8062600

[B117] Stennicke H. R. Jürgensmeier J. M. Shin H. Deveraux Q. Wolf B. B. Yang X. (1998). Pro-caspase-3 Is a Major Physiologic Target of Caspase-8. J. Biol. Chem. 273 (42), 27084–27090. 10.1074/jbc.273.42.27084 9765224

[B118] Stoll G. Ma Y. Yang H. Kepp O. Zitvogel L. Kroemer G. (2017). Pro-necrotic Molecules Impact Local Immunosurveillance in Human Breast Cancer. Oncoimmunology 6 (4), e1299302. 10.1080/2162402X.2017.1299302 28507808 PMC5414877

[B119] Sun Y. Chen P. Zhai B. Zhang M. Xiang Y. Fang J. (2020). The Emerging Role of Ferroptosis in Inflammation. Biomed. Pharmacother. 127, 110108. 10.1016/j.biopha.2020.110108 32234642

[B120] Tait S. W. Green D. R. (2010). Mitochondria and Cell Death: Outer Membrane Permeabilization and beyond. Nat. Rev. Mol. Cel Biol 11 (9), 621–632. 10.1038/nrm2952 20683470

[B121] Tamura M. Tanaka S. Fujii T. Aoki A. Komiyama H. Ezawa K. (2007). Members of a Novel Gene Family, Gsdm, Are Expressed Exclusively in the Epithelium of the Skin and Gastrointestinal Tract in a Highly Tissue-specific Manner. Genomics 89 (5), 618–629. 10.1016/j.ygeno.2007.01.003 17350798

[B122] Thongnak L. Pongchaidecha A. Lungkaphin A. (2020). Renal Lipid Metabolism and Lipotoxicity in Diabetes. Am. J. Med. Sci. 359 (2), 84–99. 10.1016/j.amjms.2019.11.004 32039770

[B123] Tinton S. A. Lefebvre V. H. Cousin O. C. Buc-Calderon P. M. (1993). Cytolytic Effects and Biochemical Changes Induced by Extracellular ATP to Isolated Hepatocytes. Biochim. Biophys. Acta 1176 (1-2), 1–6. 10.1016/0167-4889(93)90169-p 8452865

[B124] Tixeira R. Shi B. Parkes M. A. F. Hodge A. L. Caruso S. Hulett M. D. (2018). Gasdermin E Does Not Limit Apoptotic Cell Disassembly by Promoting Early Onset of Secondary Necrosis in Jurkat T Cells and THP-1 Monocytes. Front. Immunol. 9, 2842. 10.3389/fimmu.2018.02842 30564238 PMC6288192

[B125] Tomei L. D. Umansky S. R. (1998). Aging and Apoptosis Control. Neurol. Clin. 16 (3), 735–745. 10.1016/s0733-8619(05)70091-8 9666047

[B126] Tonnus W. Meyer C. Paliege A. Belavgeni A. von Mässenhausen A. Bornstein S. R. (2019). The Pathological Features of Regulated Necrosis. J. Pathol. 247 (5), 697–707. 10.1002/path.5248 30714148

[B127] Tu X. Ye X. Xie C. Chen J. Wang F. Zhong S. (2015). Combination Therapy with Chinese Medicine and ACEI/ARB for the Management of Diabetic Nephropathy: The Promise in Research Fragments. Curr. Vasc. Pharmacol. 13 (4), 526–539. 10.2174/1570161112666141014153410 25360835

[B128] Twiddy D. Cohen G. M. Macfarlane M. Cain K. (2006). Caspase-7 Is Directly Activated by the Approximately 700-kDa Apoptosome Complex and Is Released as a Stable XIAP-Caspase-7 Approximately 200-kDa Complex. J. Biol. Chem. 281 (7), 3876–3888. 10.1074/jbc.M507393200 16352606

[B129] Typiak M. Kulesza T. Rachubik P. Rogacka D. Audzeyenka I. Angielski S. (2021). Role of Klotho in Hyperglycemia: Its Levels and Effects on Fibroblast Growth Factor Receptors, Glycolysis, and Glomerular Filtration. Int. J. Mol. Sci. 22 (15), 7867. 10.3390/ijms22157867 34360633 PMC8345972

[B130] Ursini F. Maiorino M. Valente M. Ferri L. Gregolin C. (1982). Purification from Pig Liver of a Protein Which Protects Liposomes and Biomembranes from Peroxidative Degradation and Exhibits Glutathione Peroxidase Activity on Phosphatidylcholine Hydroperoxides. Biochim. Biophys. Acta 710 (2), 197–211. 10.1016/0005-2760(82)90150-3 7066358

[B131] Van Laer L. Huizing E. H. Verstreken M. van Zuijlen D. Wauters J. G. Bossuyt P. J. (1998). Nonsyndromic Hearing Impairment Is Associated with a Mutation in DFNA5. Nat. Genet. 20 (2), 194–197. 10.1038/2503 9771715

[B132] Volarevic V. Djokovic B. Jankovic M. G. Harrell C. R. Fellabaum C. Djonov V. (2019). Molecular Mechanisms of Cisplatin-Induced Nephrotoxicity: a Balance on the Knife Edge between Renoprotection and Tumor Toxicity. J. Biomed. Sci. 26 (1), 25. 10.1186/s12929-019-0518-9 30866950 PMC6417243

[B133] Wada J. Makino H. (2016). Innate Immunity in Diabetes and Diabetic Nephropathy. Nat. Rev. Nephrol. 12 (1), 13–26. 10.1038/nrneph.2015.175 26568190

[B134] Wang H. Guan J. Guan L. Yang J. Wu K. Lin Q. (2018). Further Evidence for "Gain-Of-Function" Mechanism of DFNA5 Related Hearing Loss. Sci. Rep. 8 (1), 8424. 10.1038/s41598-018-26554-7 29849037 PMC5976723

[B135] Wang H. Liu C. Zhao Y. Gao G. (2020). Mitochondria Regulation in Ferroptosis. Eur. J. Cel Biol 99 (1), 151058. 10.1016/j.ejcb.2019.151058 31810634

[B136] Wang Y. Gao W. Shi X. Ding J. Liu W. He H. (2017). Chemotherapy Drugs Induce Pyroptosis through Caspase-3 Cleavage of a Gasdermin. Nature 547 (7661), 99–103. 10.1038/nature22393 28459430

[B137] Wang Y. Yin B. Li D. Wang G. Han X. Sun X. (2018). GSDME Mediates Caspase-3-dependent Pyroptosis in Gastric Cancer. Biochem. Biophys. Res. Commun. 495 (1), 1418–1425. 10.1016/j.bbrc.2017.11.156 29183726

[B138] Wang Y. Zhang X. Wang P. Shen Y. Yuan K. Li M. (2019). Sirt3 Overexpression Alleviates Hyperglycemia-Induced Vascular Inflammation through Regulating Redox Balance, Cell Survival, and AMPK-Mediated Mitochondrial Homeostasis. J. Recept Signal. Transduct Res. 39 (4), 341–349. 10.1080/10799893.2019.1684521 31680596

[B139] Wang Y. Zhu X. Yuan S. Wen S. Liu X. Wang C. (2019). TLR4/NF-κB Signaling Induces GSDMD-Related Pyroptosis in Tubular Cells in Diabetic Kidney Disease. Front. Endocrinol. (Lausanne) 10, 603. 10.3389/fendo.2019.00603 31608008 PMC6761221

[B140] Wang Y. Kanneganti T.-D. (2021). From Pyroptosis, Apoptosis and Necroptosis to PANoptosis: A Mechanistic Compendium of Programmed Cell Death Pathways. Comput. Struct. Biotechnol. J. 19, 4641–4657. 10.1016/j.csbj.2021.07.038 34504660 PMC8405902

[B141] Wang Y. Y. Liu X. L. Zhao R. (2019). Induction of Pyroptosis and its Implications in Cancer Management. Front. Oncol. 9, 971. 10.3389/fonc.2019.00971 31616642 PMC6775187

[B142] Wang Z. Gu Z. Hou Q. Chen W. Mu D. Zhang Y. (2020). Zebrafish GSDMEb Cleavage-Gated Pyroptosis Drives Septic Acute Kidney Injury *In Vivo* . J. Immunol. 204 (7), 1929–1942. 10.4049/jimmunol.1901456 32111733

[B143] Wen S. Wang Z. H. Zhang C. X. Yang Y. Fan Q. L. (2020). Caspase-3 Promotes Diabetic Kidney Disease through Gasdermin E-Mediated Progression to Secondary Necrosis during Apoptosis. Diabetes Metab. Syndr. Obes. 13, 313–323. 10.2147/DMSO.S242136 32104028 PMC7020918

[B144] Wu C. Orozco C. Boyer J. Leglise M. Goodale J. Batalov S. (2009). BioGPS: an Extensible and Customizable portal for Querying and Organizing Gene Annotation Resources. Genome Biol. 10 (11), R130. 10.1186/gb-2009-10-11-r130 19919682 PMC3091323

[B145] Xia S. Hollingsworth L. R. Wu H. (2020). Mechanism and Regulation of Gasdermin-Mediated Cell Death. Cold Spring Harb Perspect. Biol. 12 (3), a036400. 10.1101/cshperspect.a036400 31451512 PMC7050592

[B146] Xia S. Zhang Z. Magupalli V. G. Pablo J. L. Dong Y. Vora S. M. (2021). Gasdermin D Pore Structure Reveals Preferential Release of Mature Interleukin-1. Nature 593 (7860), 607–611. 10.1038/s41586-021-03478-3 33883744 PMC8588876

[B147] Xia W. Li Y. Wu M. Jin Q. Wang Q. Li S. (2021). Gasdermin E Deficiency Attenuates Acute Kidney Injury by Inhibiting Pyroptosis and Inflammation. Cel Death Dis 12 (2), 139. 10.1038/s41419-021-03431-2 PMC7862699 33542198

[B148] Xie C. Wu W. Tang A. Luo N. Tan Y. (2019). lncRNA GAS5/miR-452-5p Reduces Oxidative Stress and Pyroptosis of High-Glucose-Stimulated Renal Tubular Cells. Diabetes Metab. Syndr. Obes. 12, 2609–2617. 10.2147/DMSO.S228654 31849505 PMC6910862

[B149] Xu W. F. Zhang Q. Ding C. J. Sun H. Y. Che Y. Huang H. (2021). Gasdermin E-Derived Caspase-3 Inhibitors Effectively Protect Mice from Acute Hepatic Failure. Acta Pharmacol. Sin 42 (1), 68–76. 10.1038/s41401-020-0434-2 32457417 PMC7921426

[B150] Xu Y. J. Zheng L. Hu Y. W. Wang Q. (2018). Pyroptosis and its Relationship to Atherosclerosis. Clin. Chim. Acta 476, 28–37. 10.1016/j.cca.2017.11.005 29129476

[B151] Yu C. Meng X. Zhang S. Zhao G. Hu L. Kong X. (2003). A 3-nucleotide Deletion in the Polypyrimidine Tract of Intron 7 of the DFNA5 Gene Causes Nonsyndromic Hearing Impairment in a Chinese Family. Genomics 82 (5), 575–579. 10.1016/s0888-7543(03)00175-7 14559215

[B152] Yuan J. Najafov A. Py B. F. (2016). Roles of Caspases in Necrotic Cell Death. Cell 167 (7), 1693–1704. 10.1016/j.cell.2016.11.047 27984721 PMC5381727

[B153] Zanoni I. Tan Y. Di Gioia M. Broggi A. Ruan J. Shi J. (2016). An Endogenous Caspase-11 Ligand Elicits Interleukin-1 Release from Living Dendritic Cells. Science 352 (6290), 1232–1236. 10.1126/science.aaf3036 27103670 PMC5111085

[B154] Zeng C. Y. Li C. G. Shu J. X. Xu L. H. Ouyang D. Y. Mai F. Y. (2019). ATP Induces Caspase-3/gasdermin E-Mediated Pyroptosis in NLRP3 Pathway-Blocked Murine Macrophages. Apoptosis 24 (9-10), 703–717. 10.1007/s10495-019-01551-x 31175486

[B155] Zeng H. Qi X. Xu X. Wu Y. (2020). TAB1 Regulates Glycolysis and Activation of Macrophages in Diabetic Nephropathy. Inflamm. Res. 69 (12), 1215–1234. 10.1007/s00011-020-01411-4 33044562 PMC7658079

[B156] Zhan J. F. Huang H. W. Huang C. Hu L. L. Xu W. W. (2020). Long Non-coding RNA NEAT1 Regulates Pyroptosis in Diabetic Nephropathy via Mediating the miR-34c/NLRP3 Axis. Kidney Blood Press. Res. 45 (4), 589–602. 10.1159/000508372 32721950

[B157] Zhang C. Wang X. Nie G. Wei Z. Pi S. Wang C. (2021). *In Vivo* assessment of Molybdenum and Cadmium Co-induce Nephrotoxicity via NLRP3/Caspase-1-Mediated Pyroptosis in Ducks. J. Inorg. Biochem. 224, 111584. 10.1016/j.jinorgbio.2021.111584 34479002

[B158] Zhang C. C. Li C. G. Wang Y. F. Xu L. H. He X. H. Zeng Q. Z. (2019). Chemotherapeutic Paclitaxel and Cisplatin Differentially Induce Pyroptosis in A549 Lung Cancer Cells via Caspase-3/GSDME Activation. Apoptosis 24 (3-4), 312–325. 10.1007/s10495-019-01515-1 30710195

[B159] Zheng Z. Li G. (2020). Mechanisms and Therapeutic Regulation of Pyroptosis in Inflammatory Diseases and Cancer. Int. J. Mol. Sci. 21 (4), 1456. 10.3390/ijms21041456 PMC7073143 32093389

[B160] Zhou B. Zhang J. Y. Liu X. S. Chen H. Z. Ai Y. L. Cheng K. (2018). Tom20 Senses Iron-Activated ROS Signaling to Promote Melanoma Cell Pyroptosis. Cel Res 28 (12), 1171–1185. 10.1038/s41422-018-0090-y PMC6274649 30287942

[B161] Zhu B. Cheng X. Jiang Y. Cheng M. Chen L. Bao J. (2020). Silencing of KCNQ1OT1 Decreases Oxidative Stress and Pyroptosis of Renal Tubular Epithelial Cells. Diabetes Metab. Syndr. Obes. 13, 365–375. 10.2147/DMSO.S225791 32104033 PMC7025682

[B162] Zuo Y. Chen L. He X. Ye Z. Li L. Liu Z. (2021). Atorvastatin Regulates MALAT1/miR-200c/NRF2 Activity to Protect against Podocyte Pyroptosis Induced by High Glucose. Diabetes Metab. Syndr. Obes. 14, 1631–1645. 10.2147/DMSO.S298950 33880049 PMC8053520

[B163] Zychlinsky A. Prevost M. C. Sansonetti P. J. (1992). Shigella Flexneri Induces Apoptosis in Infected Macrophages. Nature 358 (6382), 167–169. 10.1038/358167a0 1614548

