# Supplementary material for: Mini-Review: GSDME-Mediated Pyroptosis in Diabetic Nephropathy
Source: Front Pharmacol. 2021 Nov 16;12:780790. doi: 10.3389/fphar.2021.780790 (PMC8637879; doi:10.3389/fphar.2021.780790)
Supplement: Supplementary file 1 [file DataSheet1.pdf]

Supplemental table 1 for abbreviation in the manuscript

Full name	Abbreviation
gasdermin D	GSDMD
caspase (caspase-1)	Casp (Casp1)
diabetic nephropathy	DN
programmed cell death	PCD
gasdermin	GSDM
interleukin-1β	IL-1β
interleukin-18	IL-18
gasdermin E	GSDME
GSDME N-terminal fragment	N-GSDME
4,5-diphosphate phosphatidylinositol	PI(4,5)P2
diabetic kidney disease	DKD
diabetic nephropathy	DN
pathogen-associated molecular patterns	PAMPs
danger-associated molecular patterns	DAMPs
lipopolysaccharide	LPS
caspase activation and recruitment domains	CARD
pathogen recognition receptors	PRRs
receptor-interacting protein kinase 1	RIPK1
receptor-interacting protein kinase 3	RIPK3
mixed lineage kinase domain-like	MLKL
reactive oxygen species	ROS
glutathione	GSH
glutathione peroxidase 4	GPX4
cytochrome c	Cyto C
neutrophil elastase	NE
nomenclature committee on cell death	NCCD
regulated cell death	RCD
NOD-like Receptors	NLRs
absent in melanoma 2	AIM2
interferon-inducible protein 16	IFN16
apoptosis-associated speck-like protein containing a caspase recruitment domain	ASC
lactate dehydrogenase	LDH
N terminal domain of gasdermin (N-GSDM)	N-GSDM
C terminal domain of gasdermin (C-GSDM)	C-GSDM
sirtuin 3	SIRT3
endothelial glucocorticoid receptors	E-GRs
fibroblast growth factor receptor 1	FGFR1
podocyte-glucocorticoid receptor	P-GR
NOD-like receptor family pyrin domain containing 3	NLRP3
glomerular endothelial cells	GECs
High dose of glucose	HG
thioredoxin interacting protein	TXNIP
Toll-like receptor 4	TLR-4

Full name	Abbreviation
nucleotide binding and oligomerization domain-like receptor family pyrin domain-containing 3	NALP3
endoplasmic reticulum stress	ERS
metastasis-associated lung adenocarcinoma transcript 1	MALAT1
monosodium urate	MSU
long noncoding RNAs	lncRNAs
growth arrest specific 5	GAS5
nuclear paraspeckle assembly transcript 1	NEAT1
acute kidney injury	AKI
tubular epithelial cells	TECs
bone marrow-derived macrophages	BMDMs
